# Species diversity and spatial distribution of CL/VL vectors: assessing bioclimatic effect on expression plasticity of genes possessing vaccine properties isolated from wild-collected sand flies in endemic areas of Iran

**DOI:** 10.1186/s12879-021-06129-0

**Published:** 2021-05-19

**Authors:** Ali Bordbar, Parviz Parvizi

**Affiliations:** Molecular Systematics Laboratory, Parasitology Department, Pasteur Institute of Iran, 69 Pasteur Ave, Tehran, Iran

**Keywords:** Leishmaniasis, CL and VL vectors, Spatial distribution, Bioclimatic effect, Genes with vaccine traits, GIS modeling

## Abstract

**Background:**

Leishmaniasis is one of the ten most important neglected tropical diseases worldwide. Understanding the distribution of vectors of visceral and cutaneous leishmaniasis (VL/CL) is one of the significant strategic frameworks to control leishmaniasis. In this study, the extent of the bioclimatic variability was investigated to recognize a rigorous cartographic of the spatial distribution of VL/CL vectors as risk-maps using ArcGIS modeling system. Moreover, the effect of bioclimatic diversity on the fold change expression of genes possessing vaccine traits (SP15 and LeIF) was evaluated in each bioclimatic region using real-time PCR analysis.

**Methods:**

The Inverse Distance Weighting interpolation method was used to obtain accurate geography map in closely-related distances. Bioclimatic indices were computed and vectors spatial distribution was analyzed in ArcGIS10.3.1 system. Species biodiversity was calculated based on Shannon diversity index using Rv.3.5.3. Expression fold change of SP15 and LeIF genes was evaluated using cDNA synthesis and RT-qPCR analysis.

**Results:**

Frequency of *Phlebotomus papatasi* was predominant in plains areas of Mountainous bioclimate covering the CL hot spots. Mediterranean region was recognized as an important bioclimate harboring prevalent patterns of VL vectors. Semi-arid bioclimate was identified as a major contributing factor to up-regulate salivary-SP15 gene expression (*P* = 0.0050, *P* < 0.05). Also, Mediterranean bioclimate had considerable effect on up-regulation of *Leishmania*-LeIF gene in gravid and semi-gravid *P. papatasi* population (*P* = 0.0109, *P* < 0.05).

**Conclusions:**

The diversity and spatial distribution of CL/VL vectors associated with bioclimatic regionalization obtained in our research provide epidemiological risk maps and establish more effectively control measures against leishmaniasis. Oscillations in gene expression indicate that each gene has its own features, which are profoundly affected by bioclimatic characteristics and physiological status of sand flies. Given the efficacy of species-specific antigens for vaccine production, it is essential to consider bioclimatic factors that have a fundamental role in affecting the regulatory regions of environmentally responsive loci for genes used in vaccine design.

**Supplementary Information:**

The online version contains supplementary material available at 10.1186/s12879-021-06129-0.

## Background

Leishmaniasis is an epidemic-prone infectious and neglected tropical disease (NTD) caused by obligate intra-macrophage protozoa of the genus *Leishmania* [1]. There are three significant forms of leishmaniases including visceral (VL), cutaneous (CL, the most common), and mucocutaneous in the world [1]. *Leishmania* parasites are transmitted by the bite of *Leishmania*-infected female sand flies, which resulting in an extensive range of clinical manifestations mainly from self-healing cutaneous leishmaniasis (CL, the most common) to progressive fatal visceral leishmaniasis (VL) [1, 2]. Despite the worldwide distribution of leishmaniasis and also its significant disease burden, currently there are no commercially available human leishmaniasis vaccines delivering the necessary level of protection. Due to the lack of efficacious vaccines to treat leishmaniasis, studying the vectors is an important component of the effort to control leishmaniasis [3, 4]. Prior to the use of antigen-specific proteins of the genus *Leishmania* or the use of antigenic proteins of sand fly saliva as a vaccine target, the genetic and expression changes of salivary and *Leishmania* proteins must first be thoroughly evaluated in their natural habitats.

Leishmaniasis is climate-sensitive and linked to environmental changes, because each climate has its own characteristics that affect the distribution of sand flies and their mammalian reservoir hosts, and thus influencing their survival and population sizes [1, 2].

The geographical distribution of leishmaniasis is extended throughout widespread territories and is endemic in over 98 countries in the world, except the Australia, the Pacific Islands, and Antarctica [1, 5]. More than 95% of new CL cases occur in six countries including Afghanistan, Algeria, Brazil, Colombia, Iran, Iraq and the Syrian Arab Republic [1, 2]. Iran is one of the most important foci of leishmaniasis in all around the world, which is one of the major public health problems in more than half of the provinces [6]. Iran’s geographic location provides an appropriate climate and landscape habitat for different species of rodents and dogs as main reservoirs and sand flies as principle vectors [7].

In northeast of Iran, CL and VL were reported from all counties and five out of eight counties of Northern Khorasan Province, respectively [8]. CL cases are mostly adult men ≥15 years of age, occurring in approximately 500 to 1400 patients over a three-year period in Northern Khorasan Province [9]. North Khorasan is also known for VL cases with about 160 VL cases in infants, children and adults from 1990 to 2010 [10]. The procedure of natural transmission of *Leishmania* parasites to their mammalian hosts depends on many factors, including geographical location, climatic variability, attachment affinity of *Leishmania* parasites to the midgut of sand fly, and differentiation of *Leishmania* parasites into the infective life-cycle stage in sand flies, which is eventually transmitted by bite of the blood-sucking vectors of phlebotomine sand flies that have previously fed on an infected reservoir host [11–14] Climate diversity has a crucial impact on the changing of hosts and sand fly vectors distribution in endemic areas of leishmaniasis. Therefore, it is essential to investigate the ecology, biodiversity, adaptation to habitats, and distribution of sand flies according to environmental variables for understanding the risk of leishmaniasis transmission [15]. Developing reliable and sensitive monitoring is critical to the success of any public health program in the fight against NTDs. Hazard maps for VL/CL hotspots can be obtained by conducting the GIS-based survey [7, 16, 17]. Control measures of leishmaniasis are suitable when policy-makers and practitioners apply integrative approaches and priority intervention based on operational research, disease surveillance and health education, active case detection and effective treatment, environmental management and evaluation, control of reservoir hosts, spatial modeling, and active vector control [18, 19].

Calculating the diversity indices and comparing results is a useful method to understand the ecological status of sand flies. Evaluation of vectors biodiversity measurements, including intraspecific, interspecific, and ecosystem diversity, can be obtained at the spatial scale among natural habitats and different ecosystems [20]. Hence, determining biodiversity of sand flies in different topographic locations and studying the correlation between climatic elements and incidence of leishmaniasis can help us to establish an effective prevention method against leishmaniasis and vector-borne diseases [21–23].

In our recent research, bioinformatics analyses demonstrated that the vaccine comprising the combination of truncated forms of salivary protein from *P. papatasi* (SP15) and *Leishmania* eukaryotic initiation factor (LeIF) namely *SaLeish*, has an appropriate traits to increase both innate and specific cellular immune responses with acceptable population coverage in different endemic areas of the world [24]. The protein PpSP15 was the first identified salivary protein, and could be used in a sand fly salivary protein-based vaccine strategy as saliva-driven protective immunity against vector-transmitted leishmaniasis [25, 26]. Indeed, the immunization obtained by SP15 from *P. papatasi* and its orthologs in *P. duboscqi* (PdSP15) was sufficient for protection through specific delayed-type hypersensitivity (DTH) response with a Th1 profile in Non Human Primates (NHP) and humans [27, 28]. It has been indicated that SP15 of *P. papatasi* salivary has the ability of stimulating the dendritic cells (DC), the potent antigen presenting cells, and therefore initiating the saliva-mediated immunity conferring the induction of rapid sand flies saliva–specific Th1 immune response [25, 29]. Another protein examined in this study was LeIF, which has dual characteristics (as a Th1-type adjuvant and as a protection provider) and shared > 70% homology among old world and new world *Leishmania* spp. [24, 30]. LeIF has a high sequence similarity in lower and higher eukaryotes that is homologous and highly conserved among *Leishmania* spp. [31, 32].

LeIF protein can be used as a natural adjuvant for antigen-based vaccine strategy and results in inducing the early production of LeIF-specific Th1 cytokines, IL-12 and IFNγ in PBMC of both leishmaniasis patients and normal individuals [33, 34]. The LeIF protein has previously been used as leishmaniasis prophylactic vaccine (Leish-111f) that elicited an increase in CD4^+^ T cells and thus indicating a predominant Th1-type immune response [35]. There are few studies considering the eco-epidemiological effects according to bioclimate regionalization on the expression of sand fly salivary proteins as well as their *Leishmania* within at peak activity of sand flies in leishmaniasis endemic regions. Besides, a number of studies have merely investigated the effect of environmental response on the evolution of gene expression, regardless of the precise determination of the locations affecting the expression of genes of target associated with GIS modeling system [36].

The aims of this investigation are 1) to determine the spatial distribution of potential and principle vectors of cutaneous and visceral leishmaniasis based on bioclimatic regions in ArcGIS tool as hazardous maps in northeast of Iran, 2) to regionalize the ecological habitats of sand flies as bioclimate zones according to the environmental factors using integrative approaches, species diversity assessment and richness, 3) to calculate the similarity analysis in sand fly populations collected in different ecotopes based on Shannon-Wiener index and Jaccard coefficient based on statistical analysis of R, and 4) to evaluate changes in the expression of SP15 and *Leishmania*-LeIF genes isolated from wild-caught sand flies as immunogenic proteins in different bioclimatic regions and different physiological statuses. The immunogenic proteins SP15 from salivary glands of wild-caught sand flies and LeIF from *Leishmania*-infected sand flies are species-specific for polarization and maintenance of Th1-DTH immune response [37, 38]; therefore, they are suitable options for producing vector-parasite-based vaccine against leishmaniasis [19, 35, 39, 40].

Due to the lack of sufficient knowledge in the establishment of an integrated approach to determine the effects of climate diversity on the distribution of vectors and pathogens, the main approach of this research is to provide ground-breaking knowledge for implementing an effective intervention strategy at the regional, national, and global levels. The outcome of this investigation is also essential for future research due to its potential application in the evaluation of bioclimatic effects on the expression of appropriate immunogenic proteins for vaccine design and production.

## Methods

**Fig. 1:**
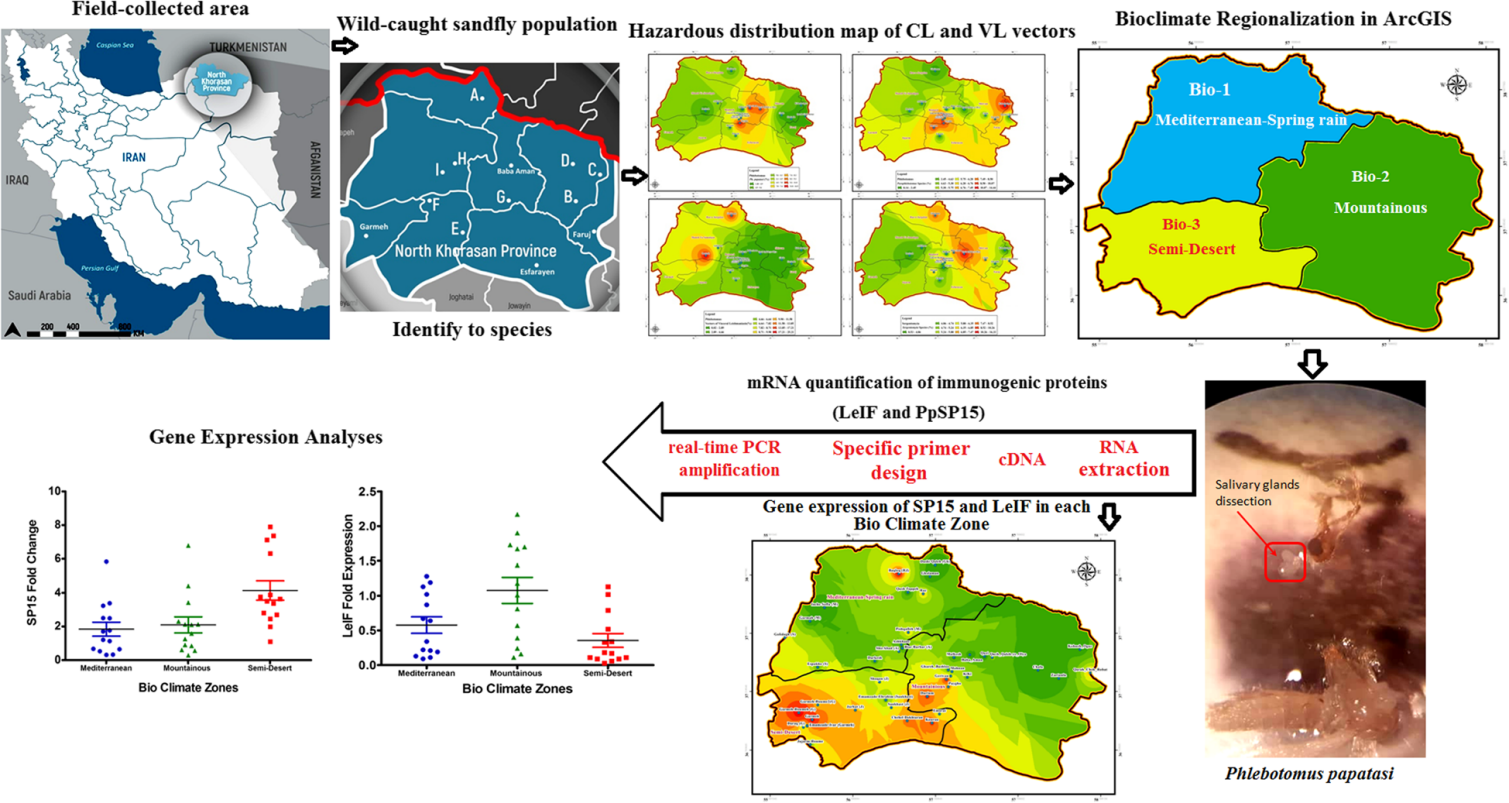
Graphical workflow represents the sequential methodology designed and performed in this study from outdoor field-working to indoor laboratory investigations using computational analysis and molecular experiments

Initially, field-working was performed based on bioclimatic zones predicted to catch native sand flies in endemic areas of leishmaniasis in Northern Khorasan Province, Iran. Spatial distribution of cutaneous leishmaniasis and visceral leishmaniasis vectors were subsequently identified and illustrated as a risk map using ArcGIS method. The effect of regionalized bioclimatic variables on the expression of two immunogenic proteins with vaccine properties (LeIF from *Leishmania* parasites and PpSP15 from sand fly saliva) was determined based on sand flies’ physiological characteristics using Real-time PCR. The fold change of the genes expression was calculated based on molecular analysis and then integrated into a bioclimatic gene expression map using ArcGIS. Figure 1 shows the outline of performed methodology and analysis.

### Study area

**Fig. 2:**
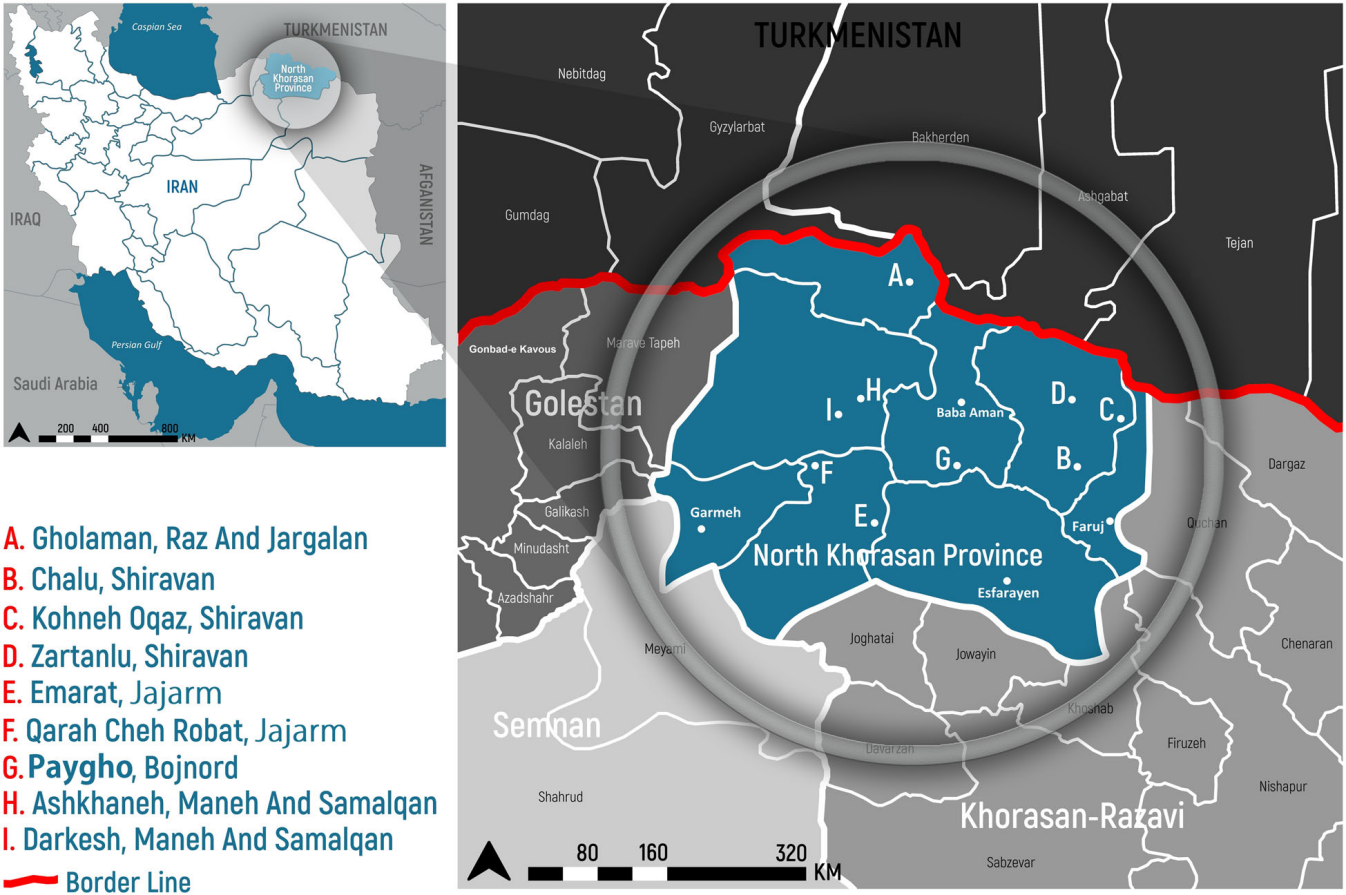
Geographical location of visceral and cutaneous leishmaniasis in north east of Iran. Main collected sites of endemic areas are marked with letters

Integrative methods were used to evaluate eco-epidemiological parameters such as bioclimatic regionalization, landscape fragmentation, and ecological niche considering vector domiciliation and their distribution in different locations of Northern Khorasan foci. Northern Khorasan province is located in north east of Iran and has been significantly inhabited by nomadic hunter-gathers since the Stone Age. Geographical location of study area (GPS coordinates) is between latitudes of 36°34 ´-38°17 ´N and longitudes of 55°52 ´-58°20 ´E. This province covers an area of 28,434 km^2^ including eight counties: Bojnord (the capital city of province), Shirvan, Esfarayen, Maneh-o Samalghan, Jajarm, Farouj, Garmeh, Raz-o Jargalan (Fig. 2). The province of Northern Khorasan is bordered by Turkmenistan to the north, Khorasan-Razavi province to the south and east, Golestan province to the west, and Semnan province to the southwest (Fig. 2). Northern Khorasan district is generally regionalized into two fractions: mountainous region, and flat areas with low elevation. Kope-Dagh in the north and Aladagh, extending of the Alborz Mountains to the south, are the two main ranges with fertile plains between the mountains that determine the favorable conditions for agriculture and pastoralism. Regarding various climate conditions, this area has a thriving culture and vegetation. The regions of Bojnord, Shirvan, Faruj, and Esfarayen are cold mountainous, while the western districts of Maneh-o Samalghan and Raz-o Jargalan are temperate and forested. Parts of the lush forest of Golestan National Park (one of the oldest national parks in the world) is located in North Khorasan Province. However, this province also has desert areas such as Jajarm, where there is a wide range of temperature fluctuations (cold winters, and relatively hot and dry summers) (Additional file 1: Figure S1a, b, c). Due to the existence of different altitudes above sea level, the entrance of various air masses and different latitudes and longitudes, southern cities have higher temperatures compared to northern regions. (Additional file 1: Figure S1). The rainfall levels were also varied in different regions of the province. Furthermore, significant rainfall amount occurs at different intensities in the province. Due to the vast mountainous areas, Northern Khorasan zone is mainly known as a cold and temperate mountainous region. The climate of the province is affected by latitude, longitude and altitude. Also, three types of basic air masses bring variety of climate conditions to Northern Khorasan region, including (1) humid western air masses that enters the province in early autumn and continues until late spring and its peak activity in the season Winter is the main cause of rainfall in the province (Additional file 1: Figure S1b), (2) Siberian cold air masses that affect the province from early autumn to early spring and cause a significant decrease in temperature (Additional file 1: Figure S1c), and (3) dry and hot air mass that enters to the province from the south in summer and increases the amount of dryness and temperature (Additional file 1: Figure S1d).

### Model variables and climatic regionalization

The De Martonne aridity index, which is used to determine the regional climate based on annual temperature and rainfall, is calculated as follows: I=P/T + 10 where (I) stands for De Martonne aridity index, P stands for annual rainfall in millimeters, and T stands for the annual temperature in °C (Fig. 2, Additional file 1: Figure S1). The maximum and minimum rainfall is observed in spring (March) and summer (June), respectively. Climatic regionalization was carried out as previously described [7] using Principal Component Analysis (PCA) as a multivariate statistical method to minimize sources available in group diversity given their unbiased character. Then, Clustering Integration Method (CIM) was applied to improve the classification accuracy. Due to vast diversity of sand fly species, even in closely distance from its sampling site, the Inverse Distance Weighting (IDW) deterministic interpolation method was used based on ArcGIS 10.3.1 analysis to specify the collected sample points in terms of sand fly dispersion and target protein expression rates (SP15 and LeIF) at the local scale. IDW is a flexible spatial interpolation method that estimates unknown values along with search distance, closest points, power setting and, barriers [41]. To obtain a geographic map in GIS, ordinary IDW parameters related to the accuracy of the values at each point was calculated according to distance: *Z*0 = $$ \frac{\sum_{i=1}^N{z}_i\cdotp {d}_i^{-n}}{\sum_{i=1}^Nd\begin{array}{c}-n\\ {}i\end{array}} $$ where *Z*0 is the estimated value of the variable z at point I, *zi* is the sample value at point I, *di* is the distance from the sample point to estimated point, *N* is the weighting coefficient based on a distance, *n* is the total number of predictions for each validation case. The Jaccardʼs similarity coefficient (JSC) is the simplest data that compares the similarity of two datasets between different locations in communities. This index always presents a value with a range from the lowest (0%) to the highest similarity (100%) between two populations [42].

### Site selection

This cross-sectional study was conducted on the peak of sand fly activity from mid-June to the late of September (2017–2018) in the most susceptible biotopes to leishmaniasis. Natural habitats of sand flies were in rural areas (vegetation, plain and deserts, and animal shelters), sylvatic-habitats of reservoir hosts (including rodents and dogs), and urban areas (human dwellings). 17 main sites along with surrounding villages were selected from different locations in Northern Khorasan province (Fig. 2, Table 1).

**Table 1:** Relative abundance and distribution of sandflies (isolated by gender) collected from main different locations of Northern Khorasan province

Climate regionalization		Mountainous																								Semi-Desert				Mediterranean						Total (%)
Main District		Bojnord												Esfarayen						Shirvan						Jajarm				Maneho-Samalghan				Razo-Jargalan		
Focal Locations		Baba Aman		Malkesh		Ghareh Bashloo		Qarah Khan Bandi		Quch Qaleh-Olya		Paygho		Dartum		Gerivan		Mahnan		Chalu		Kohneh Ogaz		Zartanlu		Emarat		Qarah Cheh Robat		Ashkhane		Darkesh		Gholaman		
Genus	Code	A		B		C		D		E		F		G		H		I		J		K		L		M		N		O		P		Q		
	Species	F	M	F	M	F	M	F	M	F	M	F	M	F	M	F	M	F	M	F	M	F	M	F	M	F	M	F	M	F	M	F	M	F	M	
*Phlebotomus*	*P. papatasi*	100	14	0	8	14	6	86	2	88	2	98	46	14	18	44	6	16	6	16	7	24	10	19	4	32	7	14	3	41	5	15	2	29	4	800 (68.55)
	*P. kandelaki*	0	0	0	0	0	0	0	0	0	0	0	0	2	0	0	0	0	0	0	0	0	0	0	0	0	0	2	0	0	0	2	0	4	0	10 (0.86)
	*P. major group*	0	0	0	0	0	0	0	0	0	0	4	0	2	0	0	0	2	0	0	0	0	0	0	0	2	0	2	1	2	0	5	0	3	0	23 (1.97)
	*P. chinensis group*	0	0	0	0	0	0	0	0	0	0	0	0	0	0	0	0	2	0	0	0	0	0	0	0	2	0	5	0	2	0	6	1	3	0	21 (1.8)
	*P. alexandri*	12	0	0	0	20	2	0	0	0	0	14	2	12	12	22	2	22	2	2	0	17	1	9	0	0	0	0	0	2	0	0	0	0	0	153 (13.11)
	*P. ansari*	0	0	0	0	2	0	0	0	0	0	0	0	0	0	0	0	0	0	0	0	0	0	0	0	0	0	0	0	0	0	0	0	0	0	2 (0.17)
	*P. caucasicus*	0	0	0	0	4	2	0	0	0	0	4	0	0	0	0	0	2	0	0	2	0	5	0	4	0	0	0	0	0	2	0	0	0	0	25 (2.14)
	*P. sergenti*	0	0	0	0	0	0	2	0	0	0	0	6	0	0	0	0	0	0	0	4	1	8	1	4	1	0	3	0	0	2	1	6	0	0	39 (3.34)
*Sergentomyia*	*S. baghdadis*	0	0	0	0	14	0	0	0	0	0	0	0	0	0	0	0	0	0	0	0	0	0	0	0	0	2	1	0	0	0	0	0	0	0	17 (1.46)
	*S. sumbarica*	4	0	0	0	0	0	4	0	0	0	0	0	4	0	0	0	2	0	0	0	0	0	0	0	0	0	0	0	0	0	0	0	0	0	14 (1.2)
	*S. dentata*	2	0	0	0	0	0	4	0	0	0	0	0	0	0	0	0	2	0	0	0	0	0	0	0	0	0	0	0	0	0	0	2	0	0	10 (0.86)
	*S. sintoni*	2	0	0	0	0	2	0	0	14	0	0	0	0	4	0	0	0	0	0	7	0	2	1	3	0	1	0	6	0	3	0	0	1	7	53 (4.54)
Total (%)		120	14	0	8	54	12	96	2	102	2	120	54	8	34	66	8	48	8	18	20	42	26	30	15	37	10	27	10	47	12	29	11	40	11	1167
		134 (11.48)		8 (0.68)		66 (5.65)		98 (8.4)		104 (8.91)		174 (14.91)		68 (5.82)		74 (6.34)		56 (4.8)		38 (3.26)		68 (5.82)		45 (3.86)		47 (4.02)		37 (3.17)		59 (5)		40 (3.42)		51 (4.37)		
		933 (79.95)																								84 (7.20)				150 (12.85)						

*F* female, *M* male, *Codes* classified for R analysis

### Sand flies collection

According to the geographical locations (altitude, latitude, and longitude) and climate conditions of cities (Fig. 2, Additional file 1: Figure S1a, b, c, d) as well as in the peak seasons of activity of sand flies (according to the incidence of leishmaniasis in each of these areas), trapping was carried out using 120 sticky oil papers, four CDC miniature light traps and, 70 funnel traps. CDC light trap is a portable sampling device for the collection of different sand fly species alive. Sticky papers are sheets of A4 paper that are painted with castor oil or pure sesame oil as adhesive and are effective at collecting sand flies. Funnel traps are installed at the entrance of rodent burrows or tree holes to capture sand flies. Catching was carried out from sunset to sunrise for five to 10 days in each location. Dead sandflies were removed from castor oil papers using a fine brush and put in sterile tubes containing 96% alcohol. Then in order to identify the species, they were kept at − 20 °C for future morphological and/or molecular experiments. Blood-fed, semi-gravid, and gravid female sand flies were collected alive by the use of aspirator, funnel traps and CDC light traps, and immediately transferred to suspended net cages using mouth aspirator. To protect captured sand flies, a cotton swab soaked in 30% sugar was placed on the top plat of the cage. It was then covered with a plastic bag containing a damp sponge to prevent moisture loss and/or overheating. Field-caught sand flies were first euthanized in water, dissected, and then identified to species based on morphological characteristics of the head and terminal genitalia under compound microscope (400×) using a systematic key [43, 44].

### RNA extraction from salivary glands and *Leishmania*-infected sand flies

Fold changes in the expression of SP15-saliva and *Leishmania*-LeIF genes were quantified in field-collected *P. papatasi* sand flies from June to mid-September at various locations in the study areas. Physiological status of sand flies was considered to determine the expression level of SP15 gene. Therefore, due to fresh-fed sand flies, have been shown a significant up-regulation of SP15 compared to un-fed ones [45], we selected only blood-fed sand flies to investigate the change in expression of SP15 transcript in different environmental and eco-epidemiological conditions in Northern Khorasan province. After definitive identification of female *P. papatasi*, salivary glands in addition to their head accessories were dissected using sterile forceps. They were then immediately resuspended in 50 μl of RNA-zole® RT solution (MRC, OH, USA), homogenized, and stored at − 20 °C. To assess the proper expression of SP15 gene, fragments isolated from 10 sand flies were pooled for total RNA isolation (Jena Bioscience, Germany).

To evaluate LeIF gene expression, sand fly-derived metacyclic promastigotes were identified by morphological features under a loop microscope. As parous sand flies (flies that lay eggs) play a crucial role in the transmission of the infective metacyclic promastigotes; fresh-fed, gravid, and semi-gravid sand flies were first examined microscopically. Then head along with the thoracic midgut forefront adjacent to the stomodeal valve (SV) and SV part of each infected sand fly were dissected based on physiological status. Also, isolation of parasites from proboscis extending to stomodeal valve of sand fly was not exempt from technical difficulties. Promastigotes obtained from aforementioned part of *P. papatasi* were counted using Neubauer chamber. The number of parasites isolated from each sand fly was approximately 10^4^ promastigotes. RNA purification was performed to obtain high-yield total RNA from *Leishmania* parasites isolated from 10 sand flies with specific status (fresh-fed, gravid, and semi-gravid) utilizing silica-gel membrane adsorption kit (Jena Bioscience, Germany, http://www.jenabioscience.com) and stored at − 80 °C until necessary. RNA quality and quantity were determined using Nanodrop (BioTek® Synergy™ HTX Multi-Mode, USA).

### cDNA synthesis

Complementary DNAs (cDNA) was synthesized as a template for quantitative Real-Time PCR (qRT PCR) using the Easy™ cDNA Synthesis Kit (Parstous Biotech, http://www.parstous.com/easy-cdna-synthesis-kit.html, Mashhad, Iran), according to the manufacturer’s instructions. Briefly, 8 μl of template RNA from each sample was mixed with kit components in RNase-free tube. After quick vortex, the prepared mixture was incubated for 10 min (min) at 25 °C, and then was incubated for one hour at 47 °C. Stopping the reaction was done by heating for 5 min at 85 °C, and chilling on ice for 10 min. To PCR or quantitative PCR, the finished RT reaction can be added up to 1/5 of the final PCR volume.

### Quantitative real-time polymerase chain reactions (RT-qPCR)

Real-time PCR reactions were performed to quantify the total amount of SP15 and LeIF genes expression from salivary gland and *Leishmania*-infected *P. papatasi* sand flies using Maxima SYBRcgreen and Rotor-Gene Q instrument (Qiagen, Germany). The qRT-PCR reactions were set up in duplicates with a total volume of 10 μl, containing 5 μl SYBR green master mix (Fermentas, UK), 0.5 μl each forward and reverse primer (0.8 μM final concentration), 1.5 μl template of cDNA and 2.5 μl Ultra-Pure DNase/RNase-Free water (Invitrogen) in 0.1 ml capillary tubes. Initial reaction was incubated for 10 min at 95 °C, followed by 40 cycles of 95 °C for 15 s (sec), annealing for 30s at 60 °C and extension for 30s at 72 °C. Fluorescent emission was measured at 530 wavelength for SYBR Green dye at the end of the elongation cycle. Expression change of SP15-salivary and *Leishmania*-LeIF gene fragments was quantified in each of the 10 sand fly groups. Each reaction was repeated four times for each gene (in duplicate in two different runs). Quantitative Real-Time PCRs were performed with a 159 bp fragment of the SP15 cDNA using specific primer pairs for SP15 (forward: SP58F, 5′-TGCATTCCCCATTGCAAATATCAG-3′, reverse: SP194R, 5′-AGCACATTCGTGCATAATTTTCC-3′), and LeIF (forward:

LF1014F, 5′-CGTCATCAACTTCGACCTGCC-3′, reverse:

LF1153R, 5′-TCGATCTGCGTGTGGTAGTG-3′) with a 159 bp fragment for the LeIF cDNA. α-tubulin gene was used as a housekeeping load control mRNA to the qPCR reactions primers Tub-P24F and Tub-P24R in *P. papatasi* sand fly [46]. All PCR experiments were followed by melting curves to check the amplicon specificity.

Different expression ratios for salivary protein and *Leishmania* genes (SP15 and LeIF) were calculated and displayed as fold changes over a control, using the 2^-ΔΔCT^ method [47]. Fold changes in gene expression were calculated by the expression 2^-ΔΔCT^, where ΔΔC_T_ = ΔC_T_ (sample) -ΔC_T_ (calibrator), ΔC_T_ = ΔC_T_ (sample) -ΔC_T_ (alpha tubulin gene), C_T_ = cycle in a statistically significant increase in the emission intensity over background. The calibrator was indicated by the average expression (mean ΔC_T_) of the newly emergent un-fed nulliparous laboratory-reared female sand flies [46]. Moreover, negative controls were considered without the templates. Fold changes were calculated for each sample in comparison with the calibrator.

### Data analysis

**Fig. 3:**
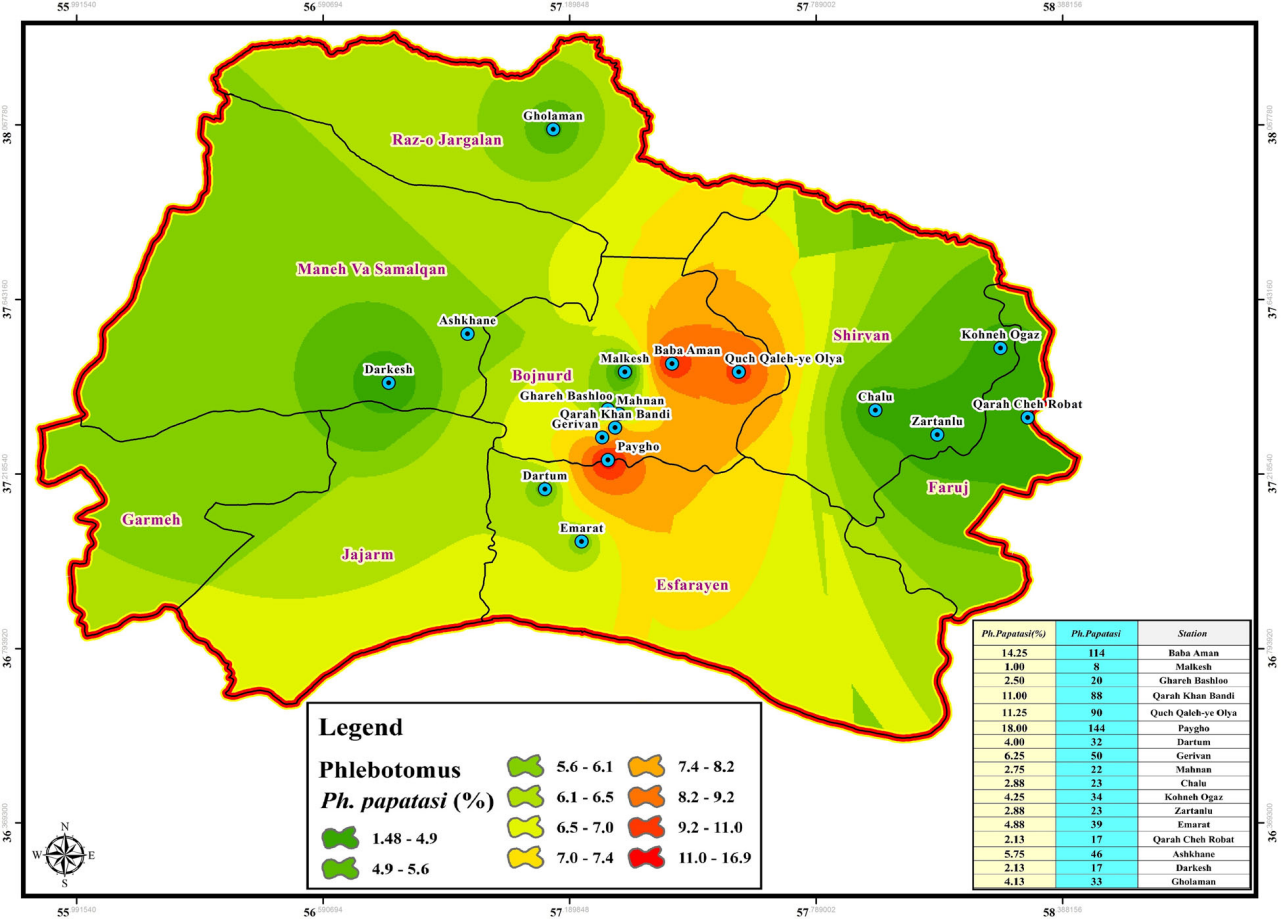
Distribution of principle vector of CL, *Phlebotomus papatasi* sand fly as hazardous map in north of Iran using IDW interpolation method in ArcGIS

**Fig. 4:**
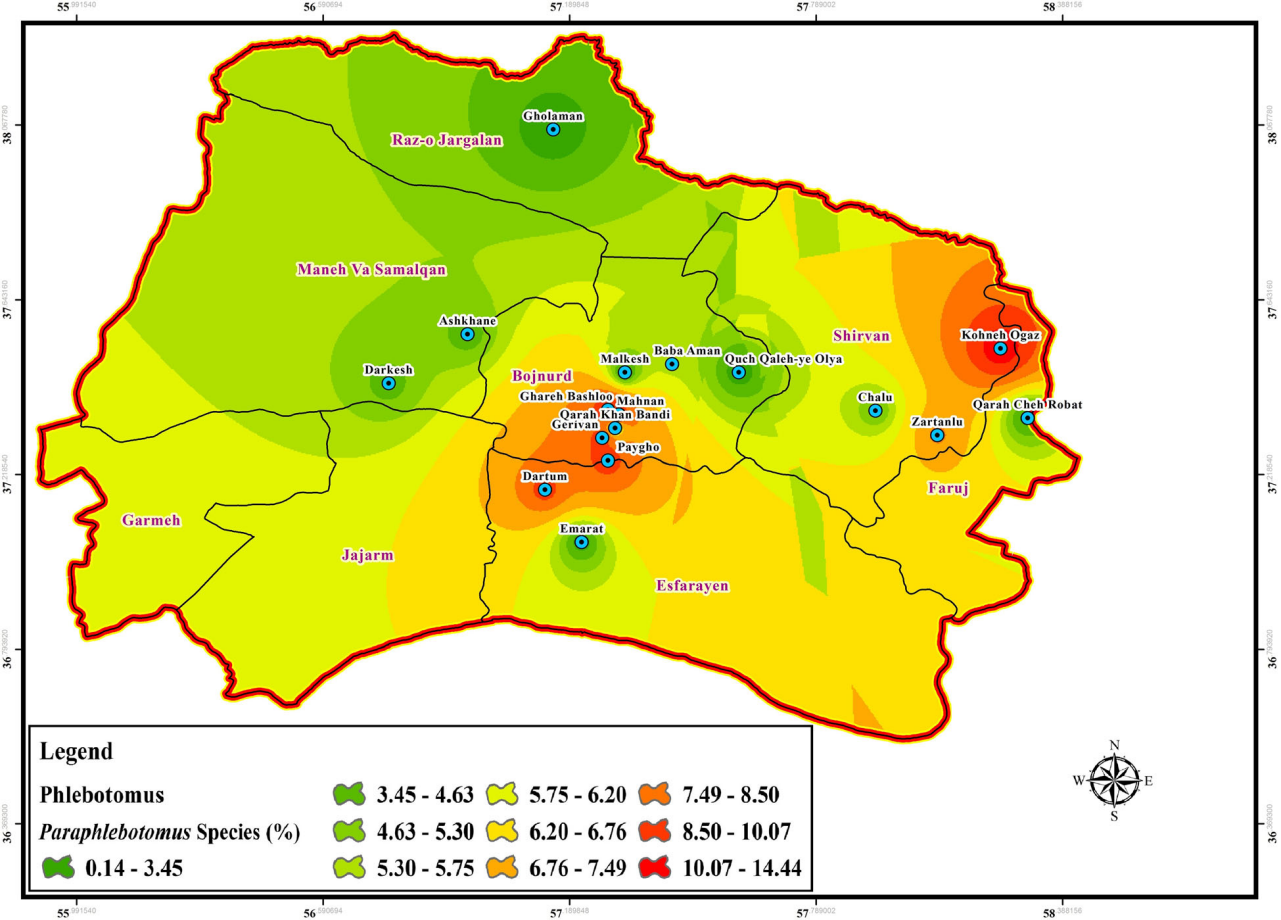
Hazardous map of *Paraphlebotomus* species, principle vectors of CL/or potential vectors of VL in north of Iran using ArcGIS

**Fig. 5:**
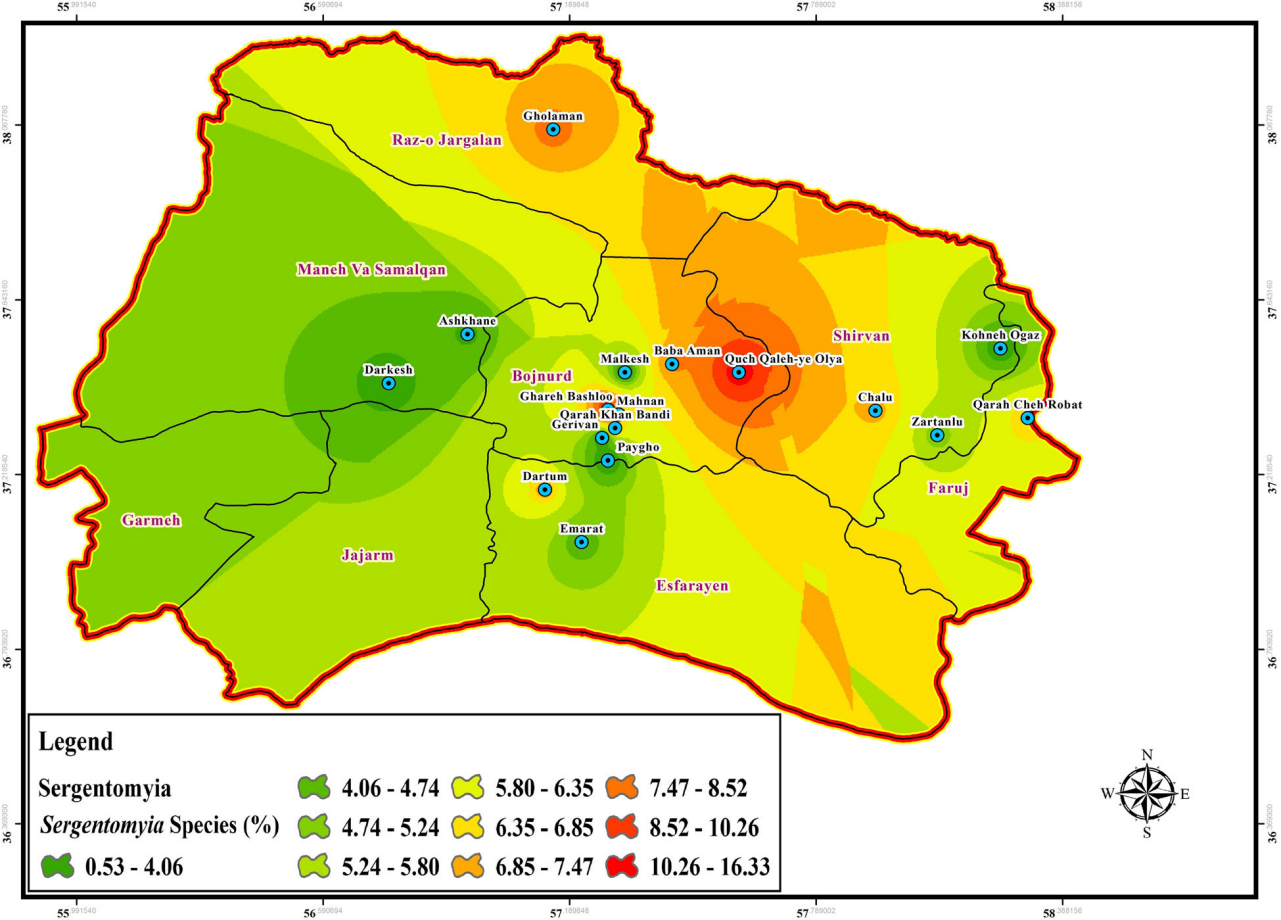
Spatial distribution of *Sergentomyia* species in northern Khorasan, Iran

**Fig. 6:**
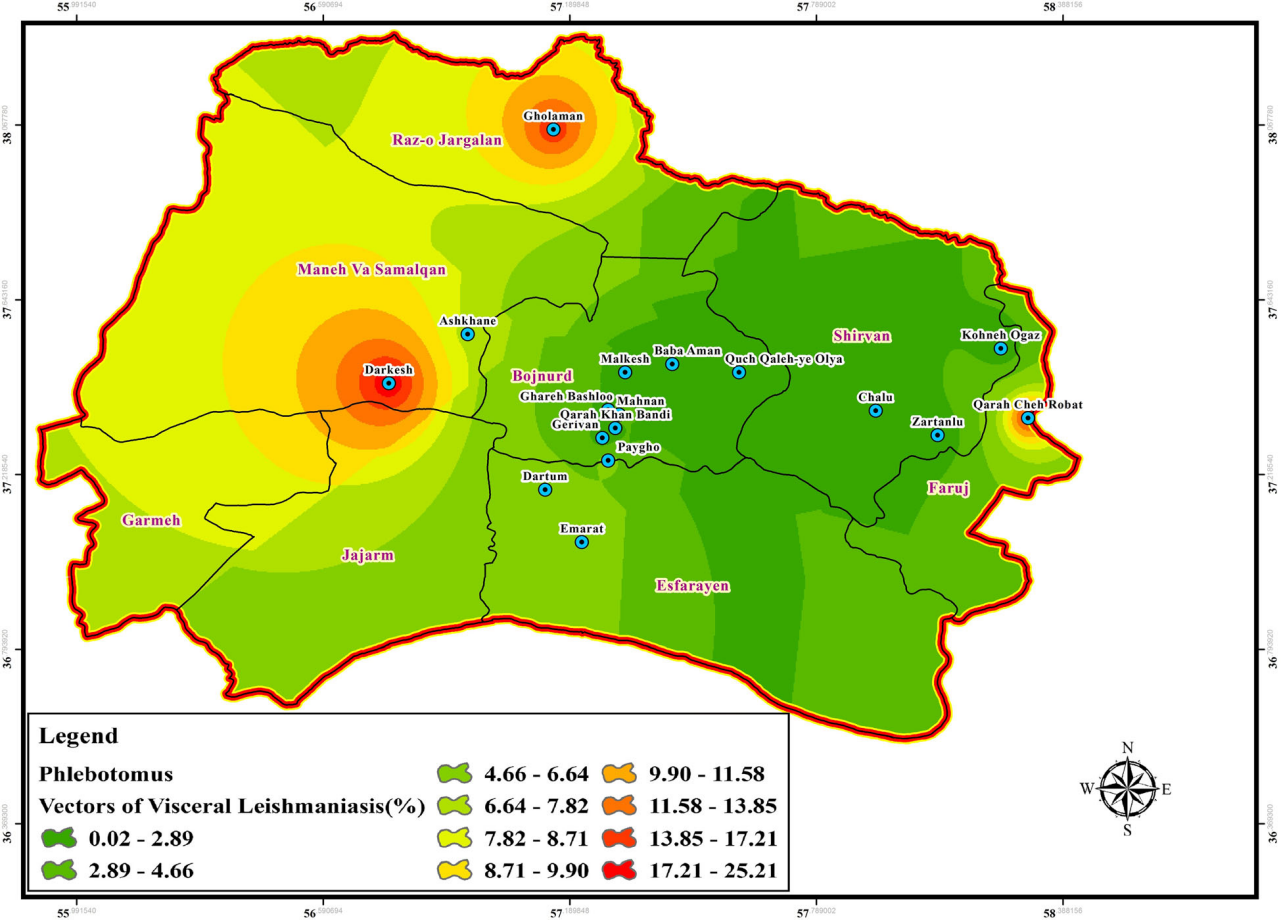
Dispersion of principle vectors of visceral leishmaniasis in north of Iran

**Fig. 7:**
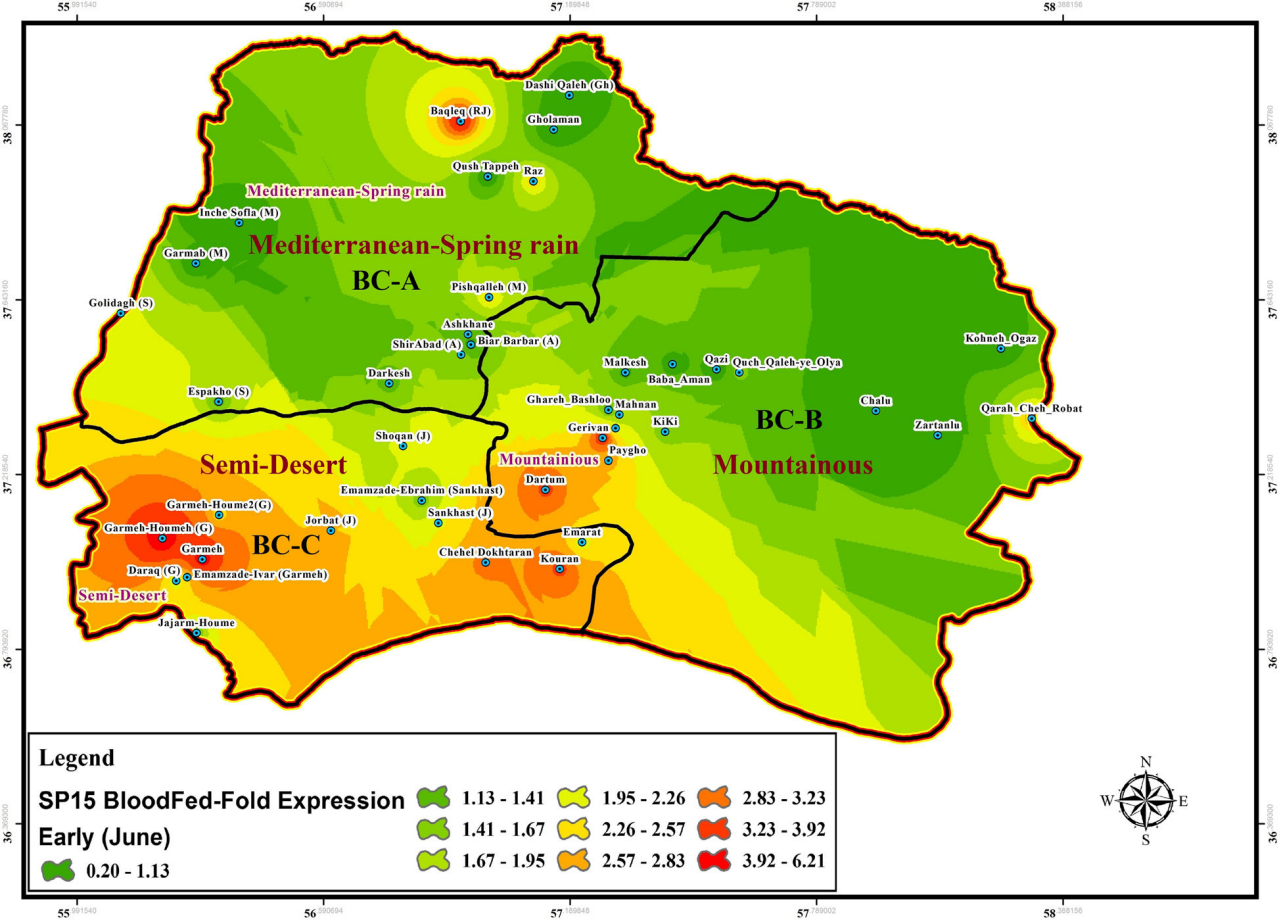
Gene expression changes of SP15 salivary protein in wild-collected *Ph. papatasi* sand flies based on bio-climate zones and time of collection, June (Early), BC: Bio Climate zones

**Fig. 8:**
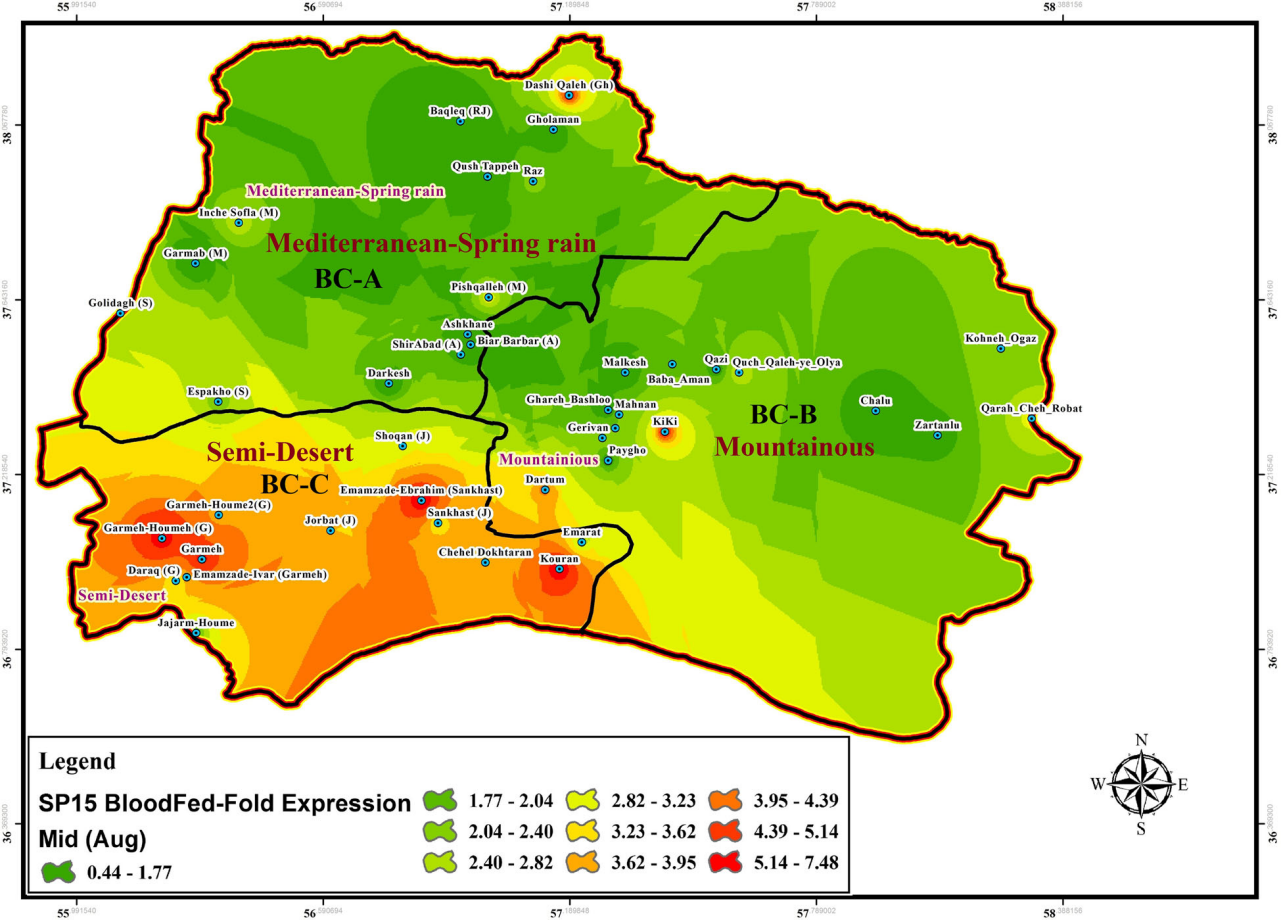
Gene expression changes of SP15 salivary protein in wild-collected *Ph. papatasi* sand flies based on bio-climate zones and time of collection, August (Mid), BC: Bio Climate areas

**Fig. 9:**
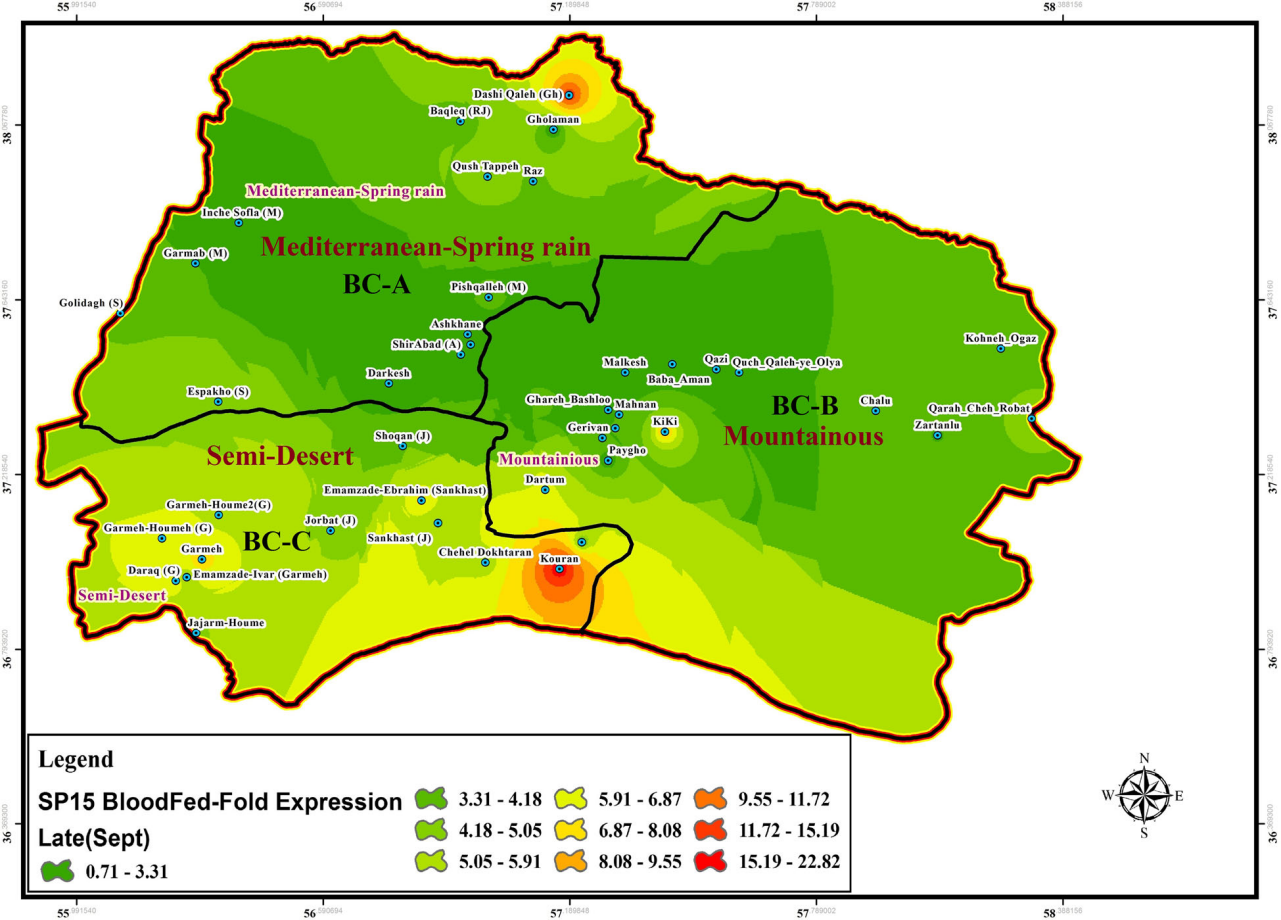
Gene expression changes of SP15 salivary protein in wild-collected *Ph. papatasi* sand flies based on bio-climate zones and time of collection, September (Late), BC: Bio Climate regions

**Fig. 10:**
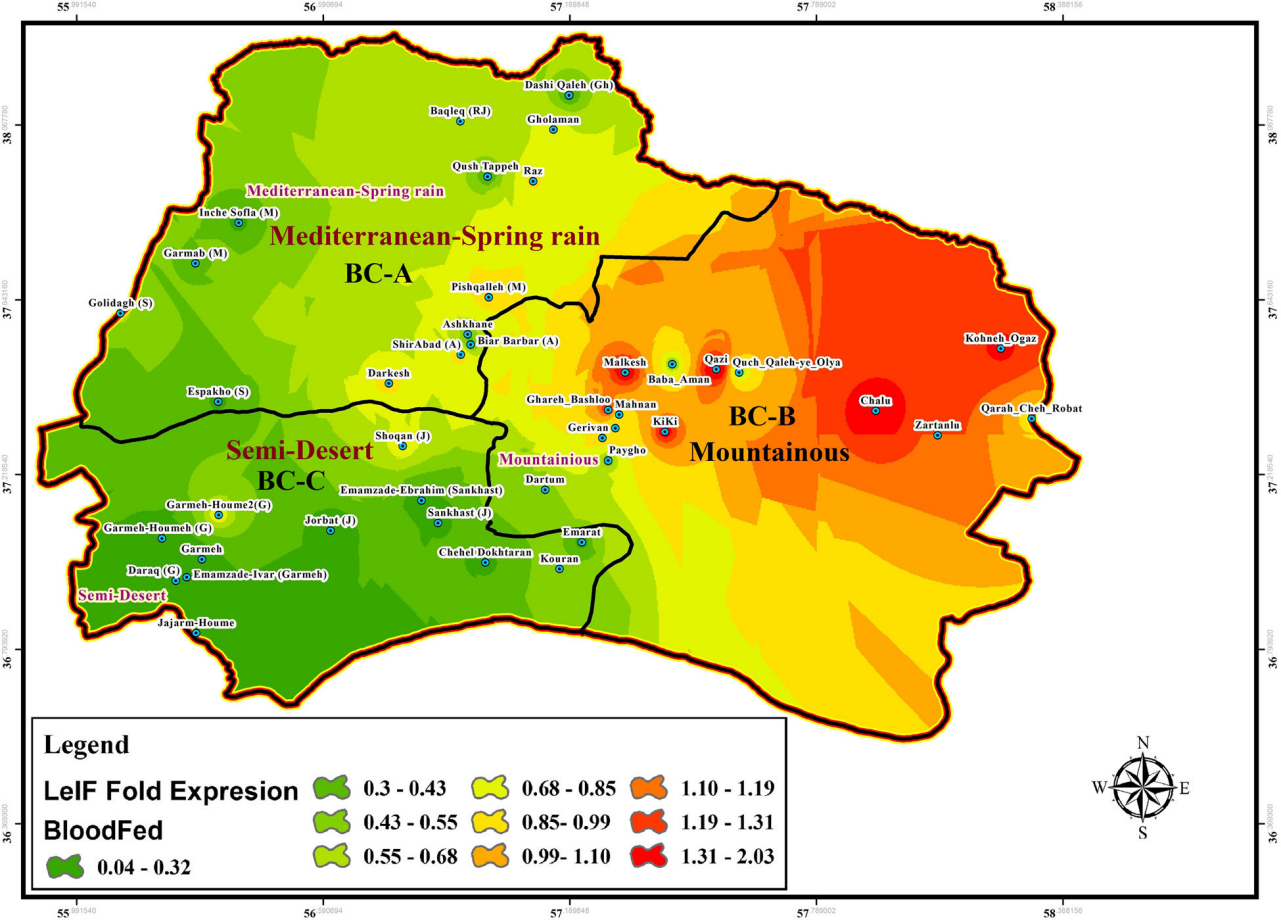
Fold change expression of *Leishmania* eukaryotic initiation factor (LeIF) gene based on “Blood-fed” physiological status of natural-collected *Ph. papatasi* sand flies in three bio-climate regions of studied area during June–September, BC: Bio Climate sites

**Fig. 11:**
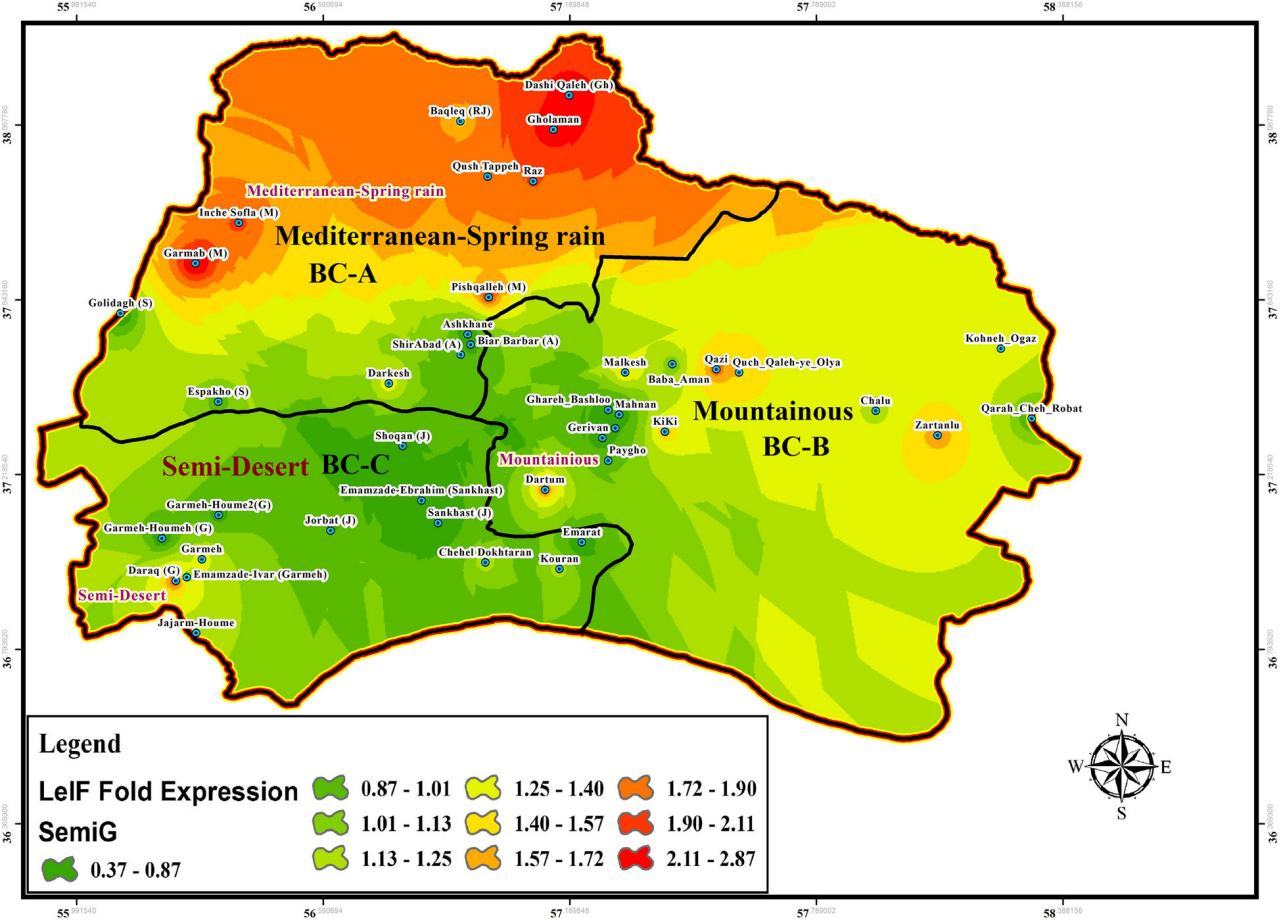
Fold change expression of LeIF gene based on “Semi-gravid” physiological status of wild-caught *Ph. papatasi* population in three bio-climate regions of studied area during June–September, BC: Bio Climate locations

**Fig. 12:**
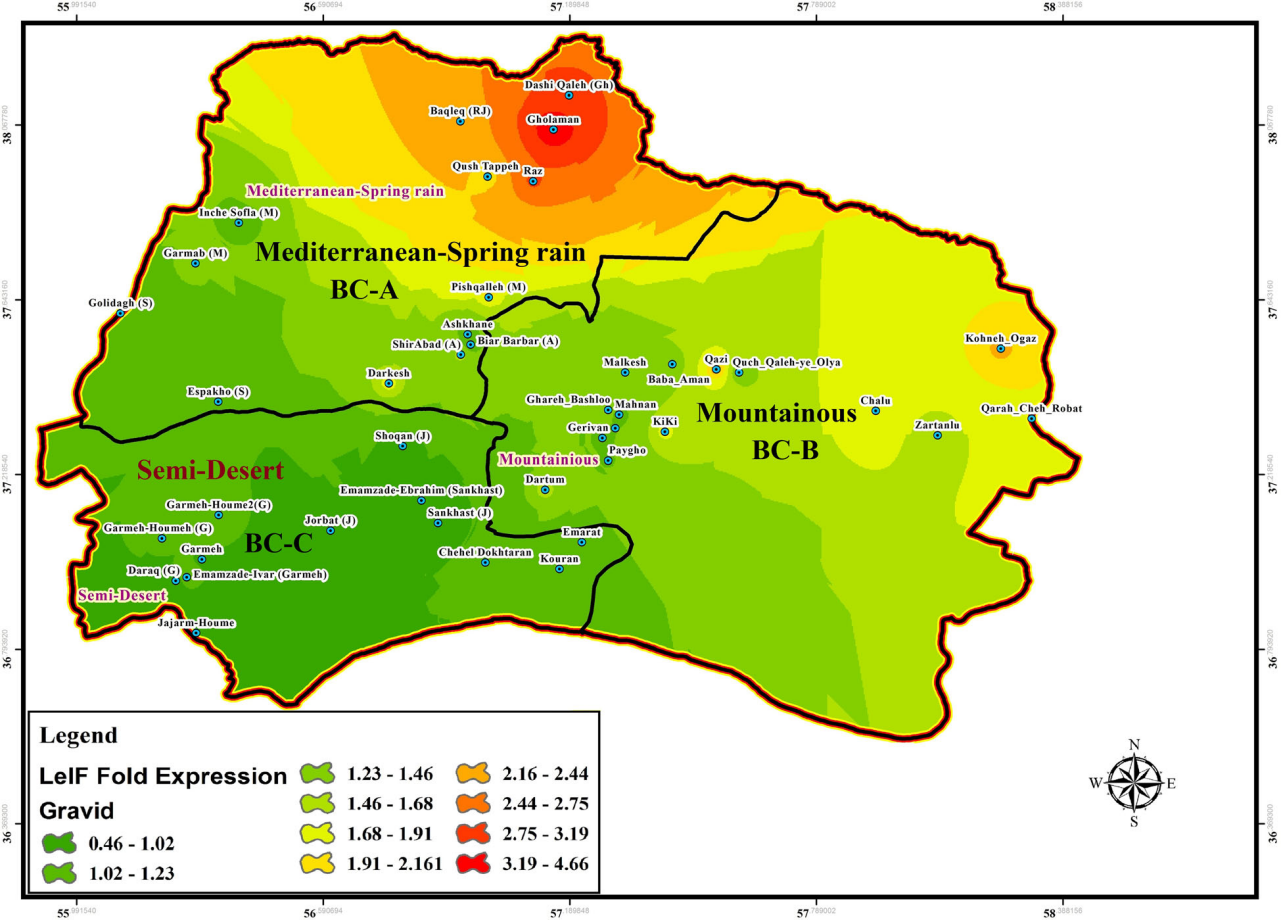
Fold change expression of LeIF gene based on “Gravid” physiological status of field-collected *Ph. papatasi* population in three bio-climate regions of studied area during June–September, BC: Bio Climate regionalized parts

**Fig. 13:**
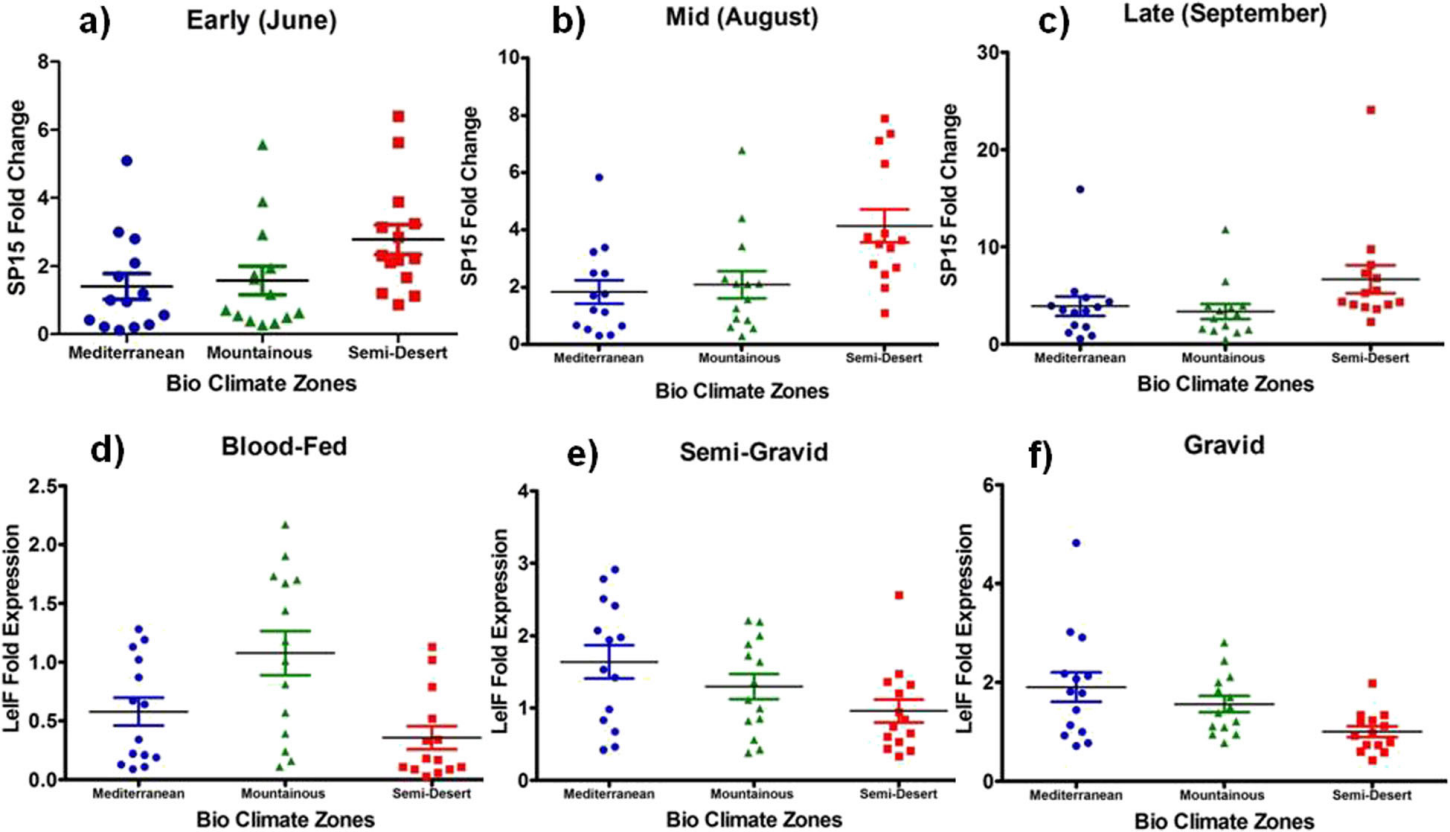
Salivary gland (SP15) and *Leishmania* (LeIF) genes expression from field-collected *P. papatasi* at northeast of Iran. Gene expression profiles were evaluated as fold changes (Y axis, SP15 and LeIF) over the calibrator, using the 2^-ΔΔCT^ method. Expression profiles of *P. papatasi* collected in June (early in the season), August (middle), and September (late) 2017–2018 are displayed for SP15 (**a**, **b**, **c**), and also for LeIF with different physiological statuses, **a**) Blood-fed, **b**) Semi-gravid, **c**) Gravid. Horizontal bars represent the expression mean values between the samples and each sample represents an individual fly. Circle, triangle and square represent each related bio-climate zones: Mediterranean, Mountainous, and Semi-dessert, respectively

Statistical analyses were carried out using the software GraphPad Prism v. 5.01 (GraphPad Software, Inc). Non-parametric Kruskal Wallis tests was applied (*P* ≤ 0.05), associated with the multiple comparison test (α = 0.05) for sampling collection. Kruskal Wallis tests was used to compare the multiple datasets with Mann-Whitney *U* tests, for accomplishing 2-way pairwise comparisons between data sets when the results for Kruskal-Wallis test were statistically significant, or for comparisons when only two data sets were present. The D’Agostino-Pearson normality test was computed the skewness and then the kurtosis, to quantify how far the distribution is from Gaussian in terms of asymmetry and shape. After that, It calculated how far each of these values differs from the value expected with a Gaussian distribution, and computed a single *P* value from the sum of these discrepancies. Species biodiversity used in this investigation is affected by two factors as: (1) number of species and (2) their abundance of distribution [42]. Species diversity was calculated based on Shannon-Wiener index, species richness and also evenness index using the Ade4 package of R v.3.5.3 software to estimate the biodiversity of sand fly species in the study areas. Species diversity is a measure of diversity within an ecological community, which encompasses both species richness and evenness of species abundance. Species richness is the number of species in a specific area or environment, whereas, species evenness refers to how close each species are in number in the similar location. Evenness is species abundance distribution and the more even this distribution is the greater the species evenness will be [48]. Computation of these indices is shown in Table 2. Also, estimation by similarity was calculated between different communities using Jaccardʼs similarity coefficient within different localities collection (Table 3). Maps and all Figs. 1, 2, 3, 4, 5, 6, 7, 8, 9, 10, 11, 12 and 13 depicted in this investigation are produced based on our own analyzed data derived from species distribution of wild-caught sand flies and gene expression levels of target genes based on climatic variables and their physiological status. The Figs. 1, 2, 3, 4, 5, 6, 7, 8, 9, 10, 11 and 12 were generated in ArcGIS tool, and statistical analyses presented in Fig. 13 are generated by GraphPad Prism tool.

**Table 2:** Biodiversity analysis for different species of sand flies in bio-climate zones of Northern Khorasan province

Main Regionalized Zones	Location	Code	*P. papatasi*			Vectors of Visceral Leishmaniasis (VL)			*Paraphlebotomus* species			*Sergentomyia* species		
			Richness (R)	Diversity (H′)	Evenness (E)	Richness (R)	Diversity (H′)	Evenness (E)	Richness (R)	Diversity (H′)	Evenness (E)	Richness (R)	Diversity (H′)	Evenness (E)
Mountainous	Baba Aman	A	0.936	0.000	0.000	0.000	0.000	0.000	0.288	0.000	0.000	1.060	1.039	0.568
	Malkesh	B	0.353	0.000	0.000	0.000	0.000	0.000	0.000	0.000	0.000	0.000	0.000	0.000
	Ghareh Bashloo	C	0.223	0.000	0.000	0.000	0.000	0.000	0.547	0.729	0.380	0.500	0.367	0.315
	Qarah Khan Bandi	D	0.106	0.000	0.000	0.000	0.000	0.000	0.707	0.000	0.000	0.707	0.693	0.721
	Quch Qaleh- Olya	E	0.105	0.000	0.000	0.000	0.000	0.000	0.000	0.000	0.000	0.267	0.000	0.000
	Paygho	F	0.083	0.000	0.000	0.500	0.000	0.000	0.588	0.925	0.495	0.000	0.000	0.000
	Dartum	G	0.176	0.000	0.000	1.000	0.693	0.721	0.204	0.000	0.000	0.707	0.693	0.721
	Gerivan	H	0.141	0.000	0.000	0.000	0.000	0.000	0.204	0.000	0.000	0.000	0.000	0.000
	Mahnan	I	0.213	0.000	0.000	1.000	0.693	0.721	0.392	0.271	0.204	1.000	0.693	0.721
	Chalu	J	0.208	0.000	0.000	0.000	0.000	0.000	1.060	1.039	0.568	0.377	0.000	0.000
	Kohneh Ogaz	K	0.171	0.000	0.000	0.000	0.000	0.000	0.530	0.970	0.528	0.707	0.000	0.000
	Zartanlu	L	0.208	0.000	0.000	0.000	0.000	0.000	0.707	1.036	0.567	0.500	0.000	0.000
Semi-desert	Emarat	M	0.160	0.000	0.000	1.000	0.693	0.721	1.000	0.000	0.000	1.154	0.636	0.641
	Qarah Cheh Robat	N	0.242	0.000	0.000	0.948	1.029	0.564	0.577	0.000	0.000	0.755	0.410	0.353
Mediterranean	Ashkhane	O	0.147	0.000	0.000	1.000	0.693	0.721	1.224	1.098	0.606	0.577	0.000	0.000
	Darkesh	P	0.242	0.000	0.000	0.801	0.922	0.548	0.377	0.000	0.000	0.707	0.000	0.000
	Gholaman	Q	0.174	0.000	0.000	0.948	1.089	0.600	0.000	0.000	0.000	0.353	0.000	0.000

*H′* Shannon-Weiner index, *R* Menhinick Richness index, *E* Shannon Evenness index

**Table 3:** Jaccard’s similarity coefficient calculated for all main localities of collecting sites of sand flies in north east of Iran

BC^a^ zones	Locality code^b^	A	B	C	D	E	F	G	H	I	J	K	L	M	N	O	P	Q
Mountainous	**A**	1	0.33	1	1	0.67	0.75	0.75	0.67	0.75	1	1	1	0.75	0.75	0.75	0.75	0.50
	**B**		1	0.33	0.33	0.50	0.25	0.25	0.50	0.25	0.33	0.33	0.33	0.25	0.25	0.25	0.25	0.33
	**C**			1	1	0.67	0.75	0.75	0.67	0.75	1	1	1	0.75	0.75	0.75	0.75	0.50
	**D**				1	0.67	0.75	0.75	0.67	0.75	1	1	1	0.75	0.75	0.75	0.75	0.50
	**E**					1	0.50	0.50	0.33	0.50	0.67	0.67	0.67	0.50	0.50	0.50	0.50	0.67
	**F**						1	1	0.50	1	0.75	0.75	0.75	1	1	1	1	0.75
	**G**							1	0.50	1	0.75	0.75	0.75	1	1	1	1	0.75
	**H**								1	0.50	0.67	0.67	0.67	0.50	0.50	0.50	0.50	0.25
	**I**									1	0.75	0.75	0.75	1	1	1	1	0.75
	**J**										1	1	1	0.75	0.75	0.75	0.75	0.50
	**K**											1	1	0.75	0.75	0.75	0.75	0.50
	**L**												1	0.75	0.75	.075	0.75	0.50
Semi-arid	**M**													1	1	1	1	0.75
	**N**														1	1	1	0.75
Mediterranean	**O**															1	1	0.75
	**P**																1	0.75
	**Q**																	1

^a^ Bio-Climate, ^b^ as shown in Table 1

## Results

In this study, climatic regionalization was performed based on computational analysis using ArcGIS modeling system to produce a risk map associated with climate implications on both cutaneous and visceral leishmaniasis in endemic areas of Iran. The impact of bioclimatic consequences on gene expression (LeIF from *Leishmania* parasites isolated from wild *Leishmania*-infected sand flies and SP15 from field-collected sand flies) was assessed by quantitative real-time PCR results, and then the fold change of the gene expression system was merged and demonstrated in a single bioclimatic-gene expression map using Arc-GIS.

### Sand fly distribution and species collections

A total of 12 species of sand flies were identified, of which 8 species belonged to the genus *Phlebotomus* and 4 species belonged to the genus *Sergentomyia* (Table 1, Additional file 2: Table S1). 1167 sandflies (283 males and 884 females) were collected in total using CDC light traps, sticky traps, aspirators and, funnel traps from three climate zones including six districts (Table 1, Additional file 2: Table S1, Additional file 3: Figure S2).

Four principal climatic elements of the study area (wind, annual precipitation, temperature, and humidity) were considered to determine bioclimatic classification and distribution of sand fly species from Northern-Khorasan province (Fig. 2, Additional file 1: Figure S1). Among aforementioned factors, annual temperature, humidity and rainfall have the greatest effects on the sand flies lives. After evaluating the effect of principle climatic factors on climatic variables, three regions were classified as homogeneous climatic zones (Table 1, Additional file 2: Table S1 (stations sheet), Additional file 3: Figure S2). The regions are as follows: (1) The Mediterranean with spring rainy zone in the northwest including Razo- Jargalan, and Maneh-o Samalqan (Table 1, Additional file 1: Figure S1, Additional file 2: Table S1). (2) The cold-mountainous region in the southeast, including the northern parts of Garmeh, in the center, including Bojnord, and in the east, including Shirvan, Faruj, and Esfarayen. (3) The low rain fall and warm-semi dry areas including Jajarm, southern and western parts of Garmeh and Esfarayen, respectively (Additional file 1: Figure S1).

The data of CL and VL vector presence (*P. papatasi*, VL species: *chinensis* group, *major* group, *P. kandelaki*, *Paraphlebotomus* species: *P. sergenti*, *P. caucasicus*, *P. ansari*, *P. alexandri*, *Sergentomyia* species: *S. sintoni*, *S. dentata*, *S. sumbarica*, *S. baghdadis*) were imported into ArcGIS tool Map. The raw data was then transferred to the visualized information to be used as a hazard map for the transmission of ZCL, ACL, and VL in each bioclimatic zones of Northern Khorasan province (Figs. 3, 4, 5, 6, Additional file 2: Table S1). In the genus *Phlebotomus*, *P. papatasi* was present in all climatic zones (*n =* 800 specimens: 68.55%). The highest frequency rate of *P. papatasi* population was found among all species of *Phlebotomus* populations. In fact, *P. papatasi* was the most abundant species in the spatial cluster of mountainous areas, especially in Baba Aman, Quch Qaleh, and Paygho where human dwellers are present in almost all ecotopes, ranging from 11.25 to 18% (Fig. 3, Table 1). The lowest population of *P. papatasi* is observed in Gholaman and Darkesh from the Mediterranean region and the farthest eastern part of mountainous regions (Fig. 3). *P. alexandri*, proven vector of VL and suspected vector of CL, is the second-most widespread species caught in mountainous climate zone (Fig. 4, Table 1, Additional file 2: Table S1 (xy sheet)). From 153 *P. alexandri*, 60.79% (*n =* 93 specimens) were caught in the sylvatic habitats of rodent burrows’ ecotope, while 39.21% *P. alexandri* (*n =* 60 specimens) were found in peridomestic animal shelters. *Paraphlebotomus* species were found only in spatial cluster of mountainous and Mediterranean bioclimatic (BC) zones (Fig. 4, Table 1). The genus *Sergentomyia* was prevalent in almost all three bioclimate habitats (Fig. 5, Table 1). However, *Sergentomyia* is more prevalent only in the spatial clusters of Mediterranean and mountainous bioclimatic zones, especially more in Gholaman, Qareh Bashloo and, Quch Qalleh-Olya with the mean range between 10.26–16.33% (Fig. 5, Table 1). *S. baghdadis* and *S. sintoni* were the predominant species of the genus *Sergentomyia* in mountainous areas (Fig. 5, Table 1, Additional file 2: Table S1 (xy sheet)) and a lower frequency is shown in green parts of Fig. 5. The presence of VL vectors was most abundant in the spatial cluster of Mediterranean region, mainly in Raz-o Jargalan and, Maneh-o Samalqan namely Gholaman and Darkesh (Fig. 6, Table 1).

### Species diversity

A series of ecological parameters and indices were used to characterize the populations of sand flies in different regions. Species diversity of sand flies was evaluated using Shannon-wiener (H′), evenness (E), and richness (S) index values along with the location of study areas (Table 2, Additional file 6: Table S4). Analysis of richness, diversity and, evenness indices of sand fly species revealed differences in communities of the three bioclimatic zones. The highest Shannon diversity and richness index was obtained from the population of *Paraphlebotomus* species with the highest rate (H′ = 1.098, R = 1.224) in the Mediterranean region compared to other bioclimatic areas (Table 2). The second richness index was related to *Sergentomyia* species (R = 1.154) in Semi-desert community, and the second Shannon diversity index was also related to *Paraphlebotomus* and *Sergentomyia* species at the same rate along with the third richness index (H′ = 1.039, R = 1.060) in a mountainous bioclimatic zone (Table 2). *P. papatasi* sand flies indicated no evenness and diversity in all collected bioclimate zones, however its richness was varied from the minimum 0.083 (Paygho) to the maximum 0.936 (Baba Aman) in Mediterranean bioclimatic ecotopes. The results of evenness analysis showed no similarity among *P. papatasi* population but our findings showed that the evenness indices for *Paraphlebotomus* species ranged from E = 0.204 in mountainous bioclimatic zone to E = 0.721 in all three bioclimatic ecotopes for vectors of visceral leishmaniasis and *Sergentomyia* species (Fig. 5, Table 2, Additional file 6: Table S4). The lack of diversity among *P. papatasi* population and visceral vectors can be caused by the extension of Alborz Mountain range (Aladagh Mountain range) as an important ecological barrier against the distribution of sand flies (Table 2). *P. alexandri* was the most abundant species among *Paraphlebotomus* population, which was mainly collected from mountainous bioclimatic areas at high altitudes (Fig. 4, Table 1, Additional file 1: Figure S1b, Additional file 2: Table S1 (xy sheet)). In fact, in agreement with previous findings in southwest of Iran [7], *P. alexandri* were caught in sylvatic habitats (Qare-Bashloo, Dartum, Gerivan and, Mahnan) near rodents ecotopes and animal shelters in the vicinity of human dwellers (Fig. 4, Additional file 2: Table S1 (xy sheet)). Also, *P. sergenti* was mostly found in rural areas (Paygho, Kohene-Oqaz, Zartanlu) in mountainous bioclimatic zone (Fig. 4, Additional file 2: Table S1 (xy sheet)). In terms of Jaccard^ʼ^s similarity coefficient for sand fly communities, the similarity of each main areas was ranged from the lowest between Malkesh (mountainous) and Paygho (mountainous), Emarat (Semi-arid), Qarahche-Robat (Semi-arid), Ashkhane (Mediterranean), Darkesh (Mediterranean) with 25% JSC index similarity to the highest among mountainous areas and, between Semi-desert (Emarat, Qarache-Robat) and Mediterranean (Ashkhane, Darkesh) bioclimatic zones with 100% JSC index (Table 3, Additional file 6: Table S4).

### SP15 and LeIF genes expression

Expression change of SP15-salivary gene isolated from uninfected female of *P. papatasi* sandflies with blood-fed status was determined using Real-time PCR at each location in three time periods in the summer (early, mid, or late) (Figs. 7, 8, 9, Table 4, Additional file 4: Table S2). The LeIF gene expression was also quantified from *Leishmania* parasites isolated from infected female of *P. papatasi* sand flies with fresh blood-fed, gravid, and semi gravid statuses in a 3-month period in the summer (Figs. 10, 11, 12, Table 4, Additional file 5 Table S3). Quantitative data for the expression profiles of SP15-saliva and *Leishmania*-LeIF genes were summarized in Table 4, Additional file 7: Table S5, based on physiological status (blood-fed, gravid and semi-gravid) and bioclimate classification (Mediterranean, mountainous and semi-arid) for female *P. papatasi* sand flies. Fold change ratio for the expression of SP15 gene varied from a minimum of 0.12 in June-early to a maximum of 24.13 in September-late in three bioclimatic regions (Figs. 7, 8, 9). Quantitative real-time PCR results showed that fold change of SP15 gene expression was ranged from 0.12 in Mediterranean bioclimate region (Gholaman) to 6.41 in Semi-arid bioclimate areas (Garmeh-Houmeh) in June-early (Fig. 7). One way ANOVA interpretation of SP15 fold expression was calculated by Kruskal-Wallis test. The results showed that the fold change expression of SP15 gene in June-early was 8.548 and the median indices indicated a significant difference for the expression of SP15 gene among wild-collected *P. papatasi* populations in all three bioclimatic regions (*P* = 0.0139, *P* < 0.05) (Fig. 7, Table 4, Additional file 7: Table S5). Although fold changes in the expression of SP15 gene indicated a marginal increase from the Mediterranean bioclimatic zone to mountainous bioclimatic zone in June-early, a significant increase was observed in the expression of SP15 gene in Semi-arid bioclimatic zone in three different time periods of collection (Fig. 13 a, b, c). Mann Whitney *U* test was used to compare the pairwise ratio of differences in SP15 gene expression by qPCR analysis between two populations of *P. papatasi* in two bioclimatic zones in early (June), mid (August), and late (September). The data obtained are summarized in Table 4, Additional file 7: Table S5. The results of fold ratios of SP15 gene expression ranged from 0.31 in the Mediterranean bioclimatic region (Darkesh) to 7.9 in the Semi-arid bioclimatic zone (holy tomb of Emamzade-Ebrahim, Sankhast County) in August-mid (Fig. 8, Additional file 4: Table S2). The Kruskal-Wallis test for SP15 fold expression was 11.19 among *P. papatasi* populations in August-mid and its median index was significant in all three bioclimatic zones (*P* = 0.0037, *P* < 0.05) (Fig. 13b, Table 4, Additional file 7: Table S5). The results of qPCR analysis showed that the expression profiles of SP15 salivary gene was ranged from 0.48 in the mountainous bioclimatic zone (Malkesh) to 24.13 in the Semi-desert bioclimatic area (Kouran district) in September-late (Fig. 9, Additional file 4: Table S2). The Kruskal-Wallis test for the expression of SP15 fold changes was 10.60 in late September, and the median was significant in all bioclimatic zones (*P* = 0.0050, *P* < 0.05) (Fig. 13c, Table 4, Additional file 7: Table S5). Notably, gene expression of salivary gland (SP15) in wild-caught *P. papatasi* revealed significant differences between humid western-wet of Mediterranean and Semi-arid (A vs C) bioclimatic zones, and it was also significant between mountainous and Semi-arid areas (B vs C) at all times of collection: June (early), August (mid) and, September (late) (Figs. 7, 8, 9, Table 4, Additional file 7: Table S5). Also, no significant change in SP15 gene expression was observed in blood-fed sand flies between Mediterranean and mountainous bioclimatic zones (A vs B) at each time (Table 4, Additional file 7: Table S5). Changes in the expression of SP15-salivary gene in blood-fed sand flies indicated a significant difference between the Semi-arid (C) and the other two bioclimatic regions. However, there was no difference between Mediterranean and mountainous bioclimatic areas (A vs B) in June-early, August-mid and, September-late (Table 4, Additional file 7: Table S5). The expression characteristics of SP15 salivary gene in three regionalized bioclimate locations are as follows: Semi-desert > Mountainous > Mediterranean (Fig. 13a, b, c, Table 4, Additional file 7: Table S5). A, B, and C are Mediterranean, mountainous, and Semi-desert bioclimatic areas, respectively, shown in Figs. 7, 8 and 9.

**Table 4:** Comparison of salivary gland and *Leishmania* gene expression levels (SP15 & LeIF) isolated from field-caught *P. papatasi* in three Bio-climate zones of Northern Khorasan province

Isolated source		Status		Climate ecotopes			Frequency of Distribution of Gene expression			*P* value (*P* < 0.05)^MW^		
				(A) Mediterranean^b^	(B) Mountainous^a^	(C) Semi arid^c^	Mean ± SEM of All 14 sites Expression Fold			All 14 sites Expression Fold		
							(A)	(B)	(C)	A vs B	A vs C	B vs C
*P. papatasi*	Salivary protein SP15 (Fold Change)	Blood-Fed	Early (June) 7 out of 14^d^	1.20	0.32	1.67	1.40429 ± 0.382118	1.585 ± 0.419459	2.77571 ± 0.434103	0.6295^N^	0.0101^S^	0.0180^S^
				0.96	0.54	3.14						
				0.42	0.49	2.23						
				1.70	1.94	2.12						
				0.20	1.73	2.85						
				0.56	2.92	5.63						
				2.80	3.89	3.88						
			Mid (Aug) 7 out of 14^d^	1.21	1.58	1.99	1.87071 ± 0.423889	2.095 ± 473,827	4.13571 ± 0.575062	0.7652^N^	0.0035^S^	0.0054^S^
				0.31	0.29	3.88						
				0.65	0.57	2.81						
				1.77	2.10	2.70						
				3.23	3.42	3.50						
				0.53	0.85	6.31						
				3.83	4.40	7.37						
			Late (Sep) 7 out of 14^d^	1.79	1.92	2.30	3.91929 ± 1.00625	3.37286 ± 0.770445	6.70214 ± 1.44592	0.5972^N^	0.0115^S^	0.0030^S^
				0.55	0.48	4.37						
				1.19	1.60	3.62						
				3.83	3.72	4.41						
				1.97	1.48	4.10						
				3.27	1.39	9.79						
				4.80	6.49	24.13						
*Leishmania parasite* (*P. papatasi*)	LeIF (Fold Change)	Gravid 7 out of 14^d^		0.770	1.090	0.920	1.90557 ± 0.298908	1.55971 ± 0.164003	0.997929 ± 108,543	0.5502^N^	0.0101^S^	0.0123^S^
				2.070	1.500	1.340						
				0.920	1.700	0.590						
				1.810	1.210	0.430						
				0.993	0.940	0.790						
				1.440	1.110	0.970						
				2.130	1.830	1.120						
		Semi-Gravid 7 out of 14^d^		0.460	0.820	0.410	1.63629 ± 0.230047	1.29536 ± 0.17331	0.956429 ± 159,147	0.3012^N^	0.0326^S^	0.1543^N^
				1.530	1.720	1.360						
				0.670	0.560	0.600						
				0.980	0.850	0.530						
				2.070	1.640	0.840						
				2.910	0.990	1.470						
				2.414	2.210	1.320						
		Blood-Fed 7 out of 14^d^		0.210	0.16	0.11	0.577857 ± 0.119376	1.07714 ± 0.188427	0.355 ± 0.0988436	0.0596^N^	0.0886^N^	0.0035^S^
				1.190	1.90	0.17						
				0.090	1.70	1.02						
				1.280	0.81	0.18						
				0.190	0.24	0.09						
				0.340	0.11	0.11						
				1.130	0.39	0.52						

^a^ Bojnord, Shirvan, Garmeh, Faruj, and Esfarayen Counties

^b^ Raz-o Jargalan, and Maneh-o Samalqan Counties

^c^ Jajarm, southern parts of Garmeh and, western areas of Esfarayen Counties, SEM: Standard Error of Mean, ^MW^, Mann-Whiteny U Test

^d^ The remaining 7 variables are shown in Supplementary 3, ^S^: Significant, ^N^: No significant

The effect of physiological status of blood meal sand flies (blood-fed, gravid, and semi-gravid) on the expression of LeIF gene was examined in natural *Leishmania* parasites isolated from infected *P. papatasi* in all three bioclimatic zones. Gravid status of *P. papatasi* showed the highest *Leishmania*-LeIF gene expression in the Mediterranean bioclimate zone (1.9 ± 0.298) (Figs. 12, 13f, Table 4, Additional file 7: Table S5). The results of RT-qPCR analysis revealed that the expression level of *Leishmania*-LeIF gene was reduced in semi-gravid status of *P. papatasi* compared to gravid in the same Mediterranean bioclimatic region (1.6 ± 0.23) (Figs. 11, 13e, Table 4, Additional file 7: Table S5). Finally once again, the gravid status of *P. papatasi* showed a slight difference in the expression of *Leishmania*-LeIF gene in mountainous bioclimate zone (1.55 ± 0.164) (Figs. 12, 13f, Table 4).

The ratio of fold changes for the expression of *Leishmania*-LeIF gene varied from at least 0.03 for the blood-fed status of *P. papatasi* in Semi-desert bioclimatic foci (Sankhast) (Figs. 10, 13d, Additional file 5: Table S3) to 4.82 as the highest fold changes of *Leishmania*-LeIF gene expression for the gravid status of *P. papatasi* in Mediterranean bioclimatic zone (Gholaman) (Figs. 12, 13f, Additional file 5: Table S3). Kruskal-Wallis test for the fold changes of LeIF gene expression was KWt = 9.046 among gravid status of *P. papatasi* populations. In fact, the median index showed a significant difference in *Leishmania*-LeIF gene expression between all populations of *P. papatasi* with gravid status in all three bioclimatic zones (*P* = 0.0109, *P* < 0.05) (Figs. 12, 13f, Table 4, Additional file 7: Table S5). Abdominal position of semi-gravid showed no significant fold changes in *Leishmania*-LeIF gene expression in wild-caught *P. papatasi* populations in all classified bioclimate ecotopes (KWt = 5.285, *P* = 0.0712, *P* < 0.05) (Figs. 11, 13e, Table 4, Additional file 7: Table S5). Statistical analysis showed that the blood-fed status of *P. papatasi* is favored in a significant fold change in *Leishmania*-LeIF gene expression in all three regionalized bioclimatic zones (KWt = 10.19, *P* = 0.0061, *P* < 0.05) (Figs. 10, 13d, Table 4, Additional file 7: Table S5).

Expression level of *Leishmania*-LeIF gene isolated from sand flies indicated a remarkable fold change in gravid status between Semi-desert and two other bioclimatic zones (C vs A and B). However, no differences in LeIF gene expression were observed among *P. papatasi* populations between Mediterranean and Mountainous (A vs B) irrigated areas from early June to late September (Figs. 12, 13f, Table 4, Additional file 7: Table S5). The transcript level of *Leishmania*-LeIF gene expression was found with a significant difference between Mediterranean and Semi-desert bioclimatic regions (A vs C) for semi-gravid sand flies, while no change in LeIF gene expression was found between Mediterranean and mountainous zones in *P. papatasi* populations (A vs B), and also, between mountainous and Semi-arid (B vs C) bioclimatic foci (Figs. 11, 13e, Table 4, Additional file 7: Table S5). Significant differences in LeIF gene expression were observed between Mediterranean and Semi-arid bioclimatic regions in *P. papatasi* populations with blood-fed status (A vs C), however, no changes in LeIF gene expression was found between Mediterranean and mountainous/semi-desert areas (A vs B-C) (Figs. 10, 13d, Table 4, Additional file 7: Table S5). Although changes in LeIF gene expression was significant between gravid sand flies of Mediterranean bioclimate and blood-fed sand flies in Semi-arid region, there was no significant difference between gravid and semi-gravid sand flies in Mediterranean area with vegetation cover (Figs. 10, 11, 12, Table 4, Additional file 7: Table S5). Nevertheless, *Leishmania*-LeIF gene expression (LeIF) in gravid and semi-gravid flies showed similar results in Mediterranean and mountainous bioclimatic regions (Fig. 13e, f, Table 4, Additional file 7: Table S5). The expression profiles of the *Leishmania*-LeIF gene isolated from field-caught *P. papatasi* sand flies are as follows: gravid of A, B and, C > semi-gravid of A, B and, C > blood-fed of A and C in three regionalized bioclimatic zones (Fig. 13d, e, f, Table 4, Additional file 7: Table S5). A, B, and C are Mediterranean, mountainous, and Semi-desert bioclimatic areas, respectively, shown in Figs. 10, 11 and 12.

## Discussion

In this study, spatial diversity and distribution of leishmaniasis vectors were investigated by applying integrative methods (PCA analysis, De Martonne aridity index and, IDW deterministic interpolation in ArcGIS system) to regionalize and also to outline the eco-epidemiological risks of VL and CL in predicted bioclimatic zones of northeastern Iran.

Rational climatic regionalization of complex bio-ecotope regions, such as endemic areas of Northern Khorasan province with three different bioclimate characteristics, is of great importance and challenging. Therefore, integrative methods including computational and molecular analysis performed in this study have been proved to be useful approaches in two ways. On the one hand, this approach facilitates the tracking of spatial patterns of vectors distribution and their related vector-borne diseases specifically in each bioclimate zone; on the other hand, it significantly enables us to evaluate the expression of immunogenic proteins in wild-caught sand flies according to identified bioclimatic patterns for leishmaniasis. The fold change expression of SP15 salivary gene from field-caught sand flies and the expression of natural *Leishmania*-LeIF gene were assessed in the population of infected sand flies in bioclimatic regions.

In line with recent reports in Iran [49], current investigation not only indicated higher diversity for specific species of sand flies in mountainous areas only in *Sergentomyia* and *Paraphlebotomus* species, but also have these results confirmed more diversity and richness of visceral leishmaniasis vectors in Semi-desert and Mediterranean bioclimatic populations (Table 2, Additional file 6: Table S4). However, no considerable diversity was observed in vectors of visceral leishmaniasis in the mountainous bioclimatic zone except in the rural areas of Dartum and Mahnan. As the zoonotic form of VL is called Mediterranean form of VL and is prevalent in endemic countries of the Mediterranean basin [50, 51], it is interesting to know that vectors of VL have a dominant distribution in Mediterranean bioclimatic zone, including Raz-o Jargalan and Manhe-o Samalqan districts (Fig. 6, Table 1). Although Maneh-o Samalqan and Raz-o Jargalan had previously been confirmed as VL endemic regions in Northern Khorasan [10], in addition to Maneh-o Samalqan, and Raz-o Jargalan in Mediterranean bioclimatic zone, we identified Semi-arid bioclimatic zone as new foci that have a pivotal role in maintaining VL vectors, especially in the northern part of Garmeh and Jajarm (Fig. 6). Recent epidemiological study has reported a high occurrence of CL cases from Garmeh, Jajarm, Bojnord, and particularly in Esfarayen as the most crucial center of CL from 2006 to 2013 [9]. In addition to the previously reported CL regions, we found that CL vectors are distributed in all three bioclimatic zones, and principle vectors of *P. papatasi* were frequently collected and identified in Mountainous bioclimatic zone, including Paygho, Baba Aman, and Quch Qaleh (Fig. 3, Table 1). Studies on the composition of sand fly species and fauna in Northern Khorasan Province showed that *P. papatasi* and *P. sergenti* are prevalent species, and *P. sergenti* is the predominant species in this area [52, 53]. But the results of our analysis revealed that *P. papatasi* is the most predominant species in Northern Khorasan province and populations of *P. alexandri*, *S. sintoni*, and *P. sergenti* are in the next distribution, respectively (Table 1). It is noteworthy that, unlike recently published data [53], our findings showed no similarity for the evenness indices in *P. papatasi* population in Northern Khorasan (Table 2). Findings of this study were also consistent with our previous results in Iran [7], which show that population of *Paraphlebotomus* species (*P. alexandri* and *P. sergenti*) tend to be colonized more in places with human inhabitants, and their peridomestic animals even in remote mountainous areas, due to anthropophilic behavior of *P. alexandri* and *P. sergenti*, (Fig. 4, Table 4, Additional file 7: Table S5). One of the important reasons for the higher frequency of visceral leishmaniasis vectors in districts of Gholaman and Darkesh compared to other mountainous bioclimatic zones can be due to the existence and development of dams along the boundary lines (Fig. 6, Additional file 2: Table S1 (xy sheet)). It is notable that each bioclimatic region has its environmental effects on the regulation of salivary protein of SP15 sand flies, which gradually reaches the highest levels of SP15 gene expression at the end of the season in the Semi-desert region (Fig. 13 a, b, c). This means that SP15 gene expression is over expressed, especially when the environment is drier and sugar sources are scarce (Figs. 7, 8, 9, Table 4, Additional file 7: Table S5) [46]. Also, in addition to blood-feeding of *P. papatasi* on mammalian hosts, feeding on various plants species as a sugar-source diet has different effects, either favorable or adverse, on life cycle [54, 55] and gonotrophic cycles [56, 57]. As a result, the diet source of sandflies is important and it increases and/or decreases the possibility of *Leishmania* transmission (depending on plant species) in irrigated or non-irrigated areas. Interestingly, our spatio-bioclimatic analyses suggests that up regulation of SP15 gene expression in Semi-arid area is a conceivable correlation of SP15 salivary protein expression with bioclimate variability depending on the type of vegetation availability and sugar sources as the main factor [58]. In fact, the expression levels of *P. papatasi* salivary gland gene (SP15) in sand flies from a drought habitat (Kouran) is higher than the expression in those flies from an irrigated area (Darkesh) late in the summer season (Fig. 9, Additional file 4: Table S2), when drought may affect the sugar content of plants [57]. Accordingly, correlation between the highest expression level of *P. papatasi* salivary SP44 gene was confirmed in arid-environment with the rarity of sugar sources in September late [46]. Moreover, aging has been proved as another physiological factor affecting the gene expression profiles. However, it is not considered as significant as genotype or sex determination in insects [59]. Also, similar to what has been observed by other researchers [45], the direct relationship was corroborated between the up-regulation of SP15 transcript and aging.

Along with other factors analyzed in this study, fluctuations in LeIF gene expression from June (early) to September (late) can be also caused by the existence of some specific plants in three points of bioclimate zones (Seim-arid, Mediterranean and, mountainous) with the different quality of sugar meals that are attractive for sugar-feeding behavior of *P. papatasi* [57]. Also, expression of genes involved in digestion of blood-meal or sugar meal in blood-fed, semi-gravid and gravid of sand fly are regulated according to the type of nutrient acquisition [60]. Therefore, the type of nutrient intake by sand flies plays an important role in the expression of *Leishmania* gene associated with distinct bioclimatic zones in the natural habitats of vectors populations. In contrast with the change in salivary-SP15 gene expression, the expression level of *Leishmania*-LeIF gene in native sand flies showed the least change in Semi-desert bioclimatic zone in all three physiological statuses of *P. papatasi* sand flies (gravid, semi-gravid, and, blood-fed) (Fig. 13d, e, f). Level of LeIF expression was significantly higher in gravid and semi-gravid sand flies compared to blood-fed sand flies. In fact, in line with recently published data [61]; our research shows that there is a decrease in LeIF expression in fresh-blood fed sandflies, and this may be due to toxic products of fresh blood-meal digestion. We also suggest that, according to Pruzinova et al. [41], since midgut binding is a key factor in the survival of *Leishmania* parasites in *P. papatasi*, therefore greater access to sugar sources in irrigated areas (Mediterranean bioclimatic zone) may be responsible for higher expression of *Leishmania* promastigote genes. Consequently, promastigote survival depends on an increase in salivary protein content [62] to exacerbate the disease in natural geographical habitats.

As leishmaniasis is considerably neglected due to its complexity of finding the pattern of vectors distribution in case of epidemiological, ecological, and bioclimatic aspects [1, 3, 5, 19], data obtained from this research can be considered as an important foundational step for the establishment of more effective control measures and the surveillance of leishmaniasis.

## Conclusions

The methodology designed and conducted in this study provides accurate information applied for case management and national strategies for controlling vector-borne diseases like leishmaniasis. The outcome of this investigation is also essential for ongoing research due to its potential use for assessing bioclimatic effects on the expression of immunogenic proteins applicable for vaccine design. Consistent with the results of this study, the impact of bioclimatic effects which effectively cause to trigger a significant increase in specific gene expression should be considered seriously. Because, as it turned out here, the effects of arid/semi-arid habitat increase the expression of some salivary gland genes, especially for SP15. Undoubtedly, the relevance of molecular biology to the mimicry of natural effect of living environment on disease agents is an underlying assumption to assist indoors lab studies. In this regard, it is possible to use the potential applications of gene drive strategy for principle vectors to regulate the expression of a specific gene at desirable function levels, and thus to control leishmaniasis or vector-borne diseases.

## Supplementary Information

**Additional file 1: Figure S1.** Northern Khorasan province was incriminated based on significant factors a) Height, b) Annual rainfall, c) Temperature, d) Humidity using deterministic spatial interpolation method in GIS.

**Additional file 2: Table S1.** Distribution of various sand fly species based on species diversity collected from three climatic zones of Northern Khorasan province, north east of Iran (2017–2018).

**Additional file 3: Figure S2.** Bioclimatic regionalization of Northern Khorasan province generated by the IDW method in ArcGIS.

**Additional file 4: Table S2.** SP15 BloodFed-Fold Expression of wild-caught *P. papatasi* in three bio climate zones at three different times.

**Additional file 5: Table S3.** LeIF Fold Expression of wild-caught *P. papatasi* in Gravid, SemiGravid, and BloodFed statuses with respect to three bio climate zones.

**Additional file 6: Table S4.** Analyses of Jaccard’s similarity index and sand fly species diversity based on Shannon-Wiener index, species richness and evenness index.

**Additional file 7: Table S5.** Analyses of 7 remaining samples related to comparison of salivary gland and *Leishmania* gene expression levels (SP15 & LeIF) isolated from field-caught *P. papatasi* in three Bioclimate zones of Northern Khorasan province.

## Data Availability

The datasets used and/or analysed during the current study are available from the corresponding author on reasonable request.

## Abbreviations

GIS: Geographic Information System
LeIF: *Leishmania* eukaryotic initiation factor
SP: Salivary protein
*SaLeish*: Saliva+*Leishmania* proteins
DTH: delayed-type hypersensitivity
DC: dendritic cells
BCz: Bio Climate zone
CL: Cutaneous leishmaniasis
VL: Visceral leishmaniasis
PCA: Principal Component Analysis
CIM: Clustering Integration Method
IDW: Inverse Distance Weighting
JSC: Jaccardʼs similarity coefficient
