# Supplementary figures and images for: Species diversity and spatial distribution of CL/VL vectors: assessing bioclimatic effect on expression plasticity of genes possessing vaccine properties isolated from wild-collected sand flies in endemic areas of Iran
Source: BMC Infect Dis. 2021 May 19;21:455. doi: 10.1186/s12879-021-06129-0 (PMC8136226; doi:10.1186/s12879-021-06129-0)

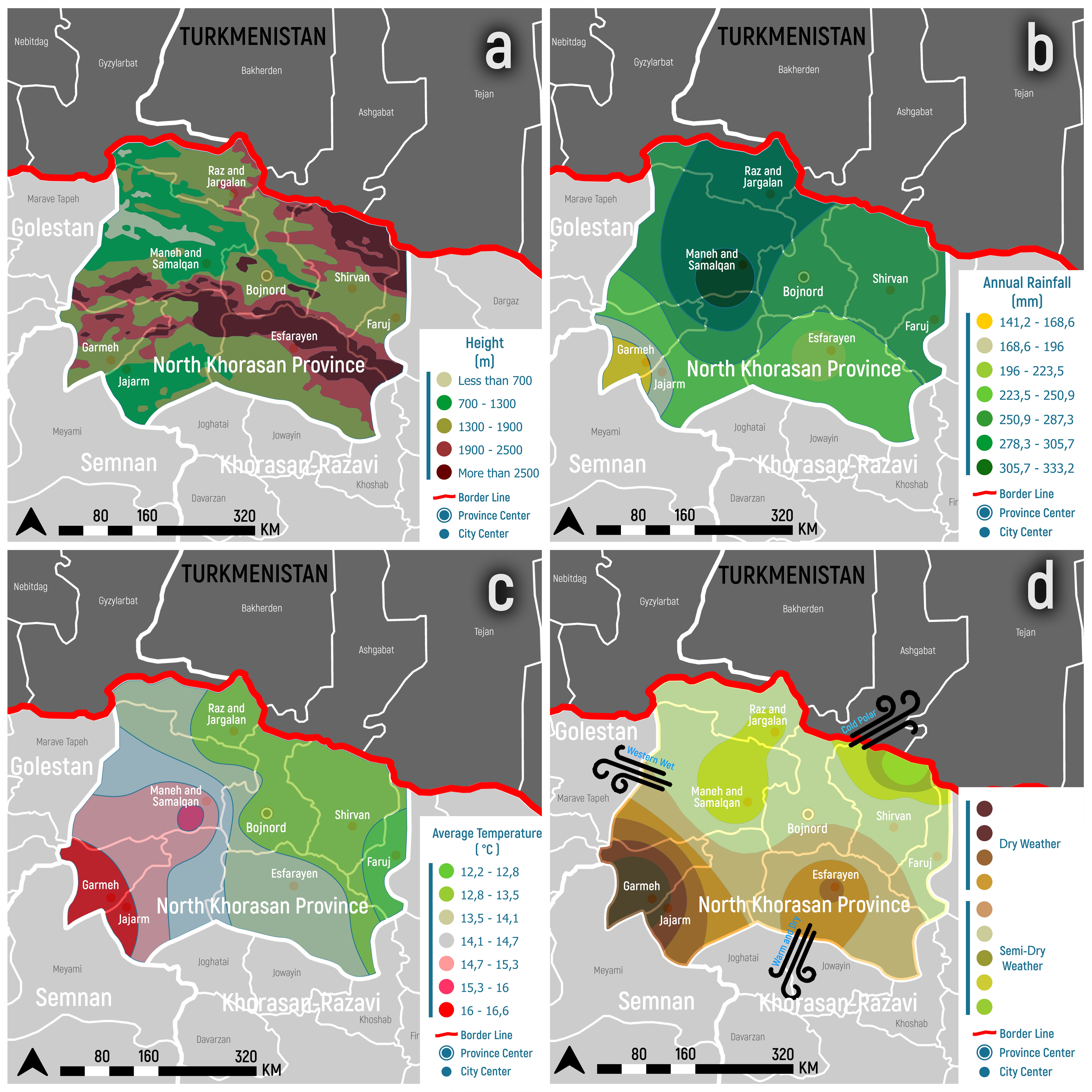

Supplement: Supplementary file 1 — Additional file 1: Figure S1. Northern Khorasan province was incriminated based on significant factors a) Height, b) Annual rainfall, c) Temperature, d) Humidity using deterministic spatial interpolation method in GIS. [file 12879_2021_6129_MOESM1_ESM.jpg]

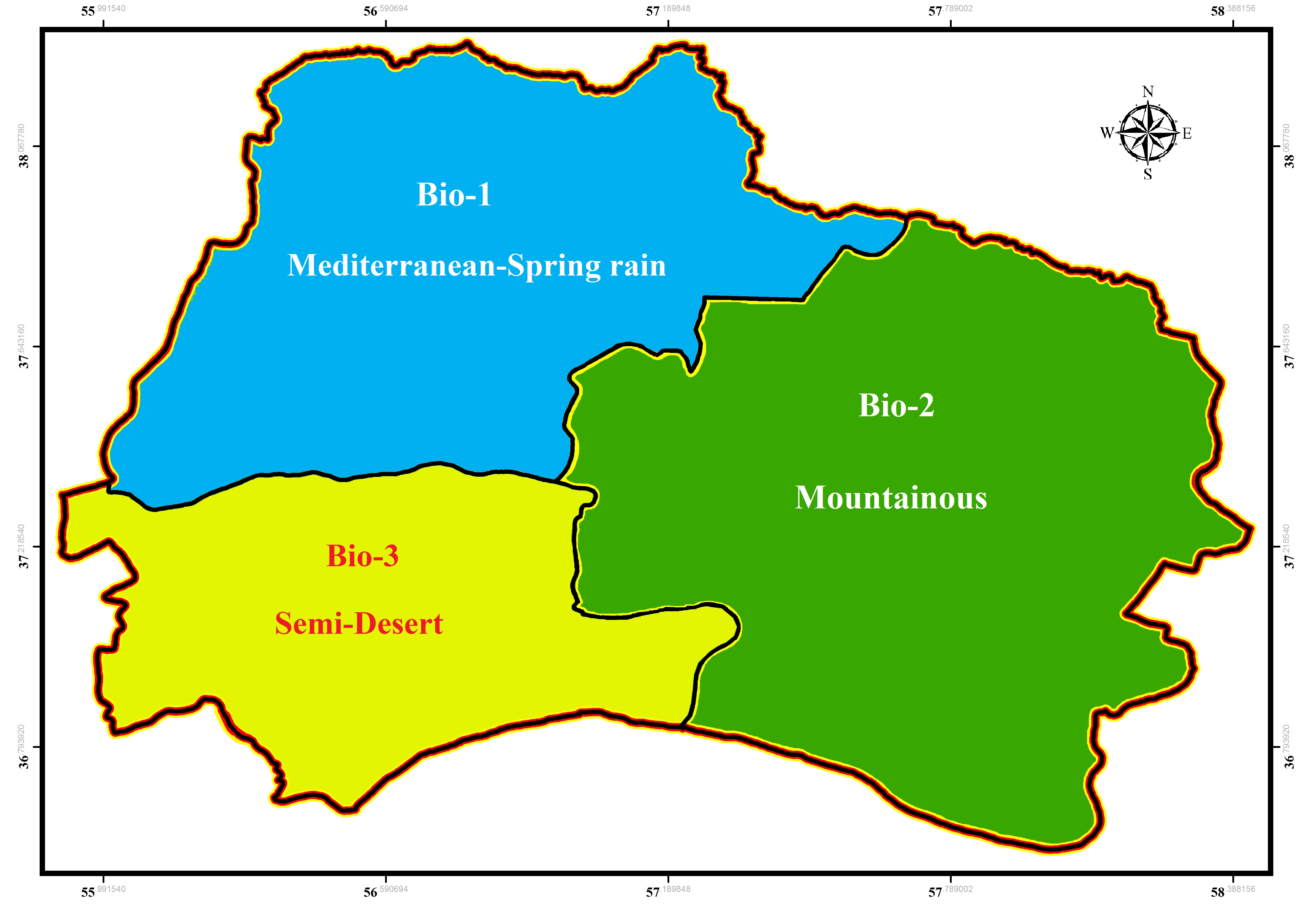

Supplement: Supplementary file 3 — Additional file 3: Figure S2. Bioclimatic regionalization of Northern Khorasan province generated by the IDW method in ArcGIS. [file 12879_2021_6129_MOESM3_ESM.jpg]
