# Supplementary material for: Species diversity and spatial distribution of CL/VL vectors: assessing bioclimatic effect on expression plasticity of genes possessing vaccine properties isolated from wild-collected sand flies in endemic areas of Iran
Source: BMC Infect Dis. 2021 May 19;21:455. doi: 10.1186/s12879-021-06129-0 (PMC8136226; doi:10.1186/s12879-021-06129-0)
Supplement: Supplementary file 7 — Additional file 7: Table S5. Analyses of 7 remaining samples related to comparison of salivary gland and Leishmania gene expression levels (SP15 & LeIF) isolated from field-caught P. papatasi in three Bioclimate zones of Northern Khorasan province. [file 12879_2021_6129_MOESM7_ESM.docx]

| Isolated source | | Status | | Climate ecotopes | | | Frequency of Distribution | | | P value (*P* < 0.05)^MW^ | | |
| --- | --- | --- | --- | --- | --- | --- | --- | --- | --- | --- | --- | --- |
|  |  |  |  | (A) Mediterranean^**^ | (B) Mountainous^*^ | (C)  Semi arid• | Mean ± SEM of All 14 Expression Fold | | | All 14 Expression Fold | | |
|  |  |  |  |  |  |  | (A) | (B) | (C) | A vs B | A vs C | B vs C |
| *Ph. papatasi* | Salivary protein SP15 (Fold Change) | Fed | Early (June)  7 remaining samples | 1.00 | 5.57 | 1.20 | 1.40429  ± 0.382118 | 1.585 ± 0.419459 | 2.77571 ± 0.434103 | 0.6295^N^ | 0.0101^S^ | 0.0180^S^ |
|  |  |  |  | 2.10 | 1.62 | 6.41 |  |  |  |  |  |  |
|  |  |  |  | 5.10 | 0.27 | 2.18 |  |  |  |  |  |  |
|  |  |  |  | 3.00 | 0.71 | 3.24 |  |  |  |  |  |  |
|  |  |  |  | 0.22 | 0.63 | 0.87 |  |  |  |  |  |  |
|  |  |  |  | 0.28 | 1.17 | 1.12 |  |  |  |  |  |  |
|  |  |  |  | 0.12 | 0.39 | 2.32 |  |  |  |  |  |  |
|  |  |  | Mid (Aug)  7 remaining samples | 1.13 | 2.12 | 2.45 | 1.87071 ± 0.423889 | 2.095 ± 473827 | 4.13571 ± 0.575062 | 0.7652^N^ | 0.0035^S^ | 0.0054^S^ |
|  |  |  |  | 2.49 | 2.30 | 7.12 |  |  |  |  |  |  |
|  |  |  |  | 0.67 | 0.61 | 3.64 |  |  |  |  |  |  |
|  |  |  |  | 2.50 | 2.13 | 3.37 |  |  |  |  |  |  |
|  |  |  |  | 1.13 | 1.26 | 1.10 |  |  |  |  |  |  |
|  |  |  |  | 5.83 | 6.79 | 7.90 |  |  |  |  |  |  |
|  |  |  |  | 0.33 | 0.91 | 3.76 |  |  |  |  |  |  |
|  |  |  | Late (Sep)  7 remaining samples | 4.37 | 2.64 | 6.83 | 3.91929  ± 1.00625 | 3.37286 ± 0.770445 | 6.70214 ± 1.44592 | 0.5972^N^ | 0.0115^S^ | 0.0030^S^ |
|  |  |  |  | 3.53 | 3.94 | 7.33 |  |  |  |  |  |  |
|  |  |  |  | 3.41 | 4.00 | 5.28 |  |  |  |  |  |  |
|  |  |  |  | 3.96 | 3.44 | 5.57 |  |  |  |  |  |  |
|  |  |  |  | 5.42 | 3.12 | 3.82 |  |  |  |  |  |  |
|  |  |  |  | 15.91 | 11.80 | 8.17 |  |  |  |  |  |  |
|  |  |  |  | 0.87 | 1.20 | 4.11 |  |  |  |  |  |  |
| *Leishmania parasite* (*Ph. papatasi*) | LeIF (Fold Change) | Gravid  7 remaining samples | | 1.130 | 0.945 | 0.736 | 1.90557 ±0.298908 | 1.55971  ± 0.164003 | 0.997929  ± 108543 | 0.5502^N^ | 0.0101^S^ | 0.0123^S^ |
|  |  |  |  | 0.710 | 0.770 | 1.235 |  |  |  |  |  |  |
|  |  |  |  | 2.175 | 2.000 | 1.190 |  |  |  |  |  |  |
|  |  |  |  | 3.020 | 2.440 | 1.980 |  |  |  |  |  |  |
|  |  |  |  | 1.780 | 1.380 | 0.600 |  |  |  |  |  |  |
|  |  |  |  | 2.910 | 2.111 | 0.730 |  |  |  |  |  |  |
|  |  |  |  | 4.820 | 2.810 | 1.340 |  |  |  |  |  |  |
|  |  | Semi-Gravid  7 remaining samples | | 0.830 | 0.425 | 2.560 | 1.63629  ± 0.230047 | 1.29536  ± 0.17331 | 0.956429  ± 159147 | 0.3012^N^ | 0.0326^S^ | 0.1543^N^ |
|  |  |  |  | 0.419 | 0.380 | 0.435 |  |  |  |  |  |  |
|  |  |  |  | 1.420 | 1.110 | 0.650 |  |  |  |  |  |  |
|  |  |  |  | 1.975 | 1.360 | 0.740 |  |  |  |  |  |  |
|  |  |  |  | 1.940 | 1.880 | 1.200 |  |  |  |  |  |  |
|  |  |  |  | 2.510 | 2.000 | 0.335 |  |  |  |  |  |  |
|  |  |  |  | 2.780 | 2.190 | 0.940 |  |  |  |  |  |  |
|  |  | Blood-Fed  7 remaining samples | | 0.220 | 1.01 | 0.09 | 0.577857 ± 0.119376 | 1.07714  ± 0.188427 | 0.355  ± 0.0988436 | 0.0596^N^ | 0.0886^N^ | 0.0035^S^ |
|  |  |  |  | 0.640 | 0.57 | 0.33 |  |  |  |  |  |  |
|  |  |  |  | 0.670 | 1.67 | 1.13 |  |  |  |  |  |  |
|  |  |  |  | 1.020 | 1.44 | 0.79 |  |  |  |  |  |  |
|  |  |  |  | 0.130 | 1.18 | 0.06 |  |  |  |  |  |  |
|  |  |  |  | 0.110 | 1.73 | 0.03 |  |  |  |  |  |  |
|  |  |  |  | 0.870 | 2.17 | 0.34 |  |  |  |  |  |  |

**Additional file 7: Table S5.** Analyses of 7 remaining samples related to comparison of salivary gland and Leishmania gene expression levels (SP15 & LeIF) isolated from field-caught *P. papatasi* in three Bio-climate zones of Northern Khorasan province.

^*^ Bojnord, Shirvan, Garmeh, Faruj, and Esfarayen Counties.

^**^ Raz-o Jargalan, and Maneh-o Samalqan Counties.

• Jajarm County, SEM: Standard Error of Mean, ^MW^, Mann-Whiteny U Test

^S^: Significant, ^N^: No significant
